# A Metabolite of *Pseudomonas* Triggers Prophage-Selective Lysogenic to Lytic Conversion in *Staphylococcus aureus*

**DOI:** 10.1021/jacs.1c01275

**Published:** 2021-05-12

**Authors:** Magdalena Jancheva, Thomas Böttcher

**Affiliations:** † Department of Chemistry, Konstanz Research School Chemical Biology, Zukunftskolleg, University of Konstanz, 78457 Konstanz, Germany; ‡ Faculty of Chemistry, Department of Biological Chemistry & Centre for Microbiology and Environmental Systems Science, University of Vienna, 1090 Vienna, Austria

## Abstract

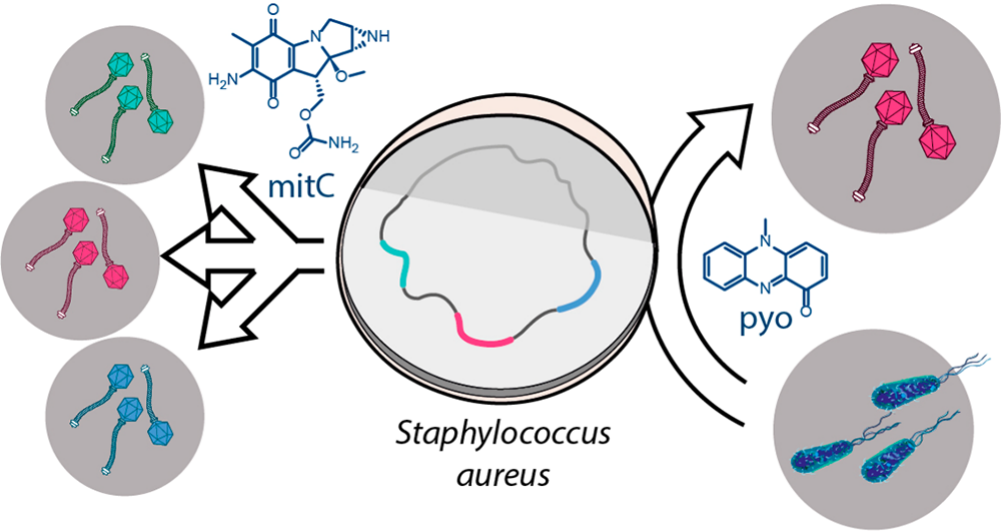

Bacteriophages have major impact on their microbial hosts and shape entire microbial communities. The majority of these phages are latent and reside as prophages integrated in the genomes of their microbial hosts. A variety of intricate regulatory systems determine the switch from a lysogenic to lytic life style, but so far strategies are lacking to selectively control prophage induction by small molecules. Here we show that *Pseudomonas aeruginosa* deploys a trigger factor to hijack the lysogenic to lytic switch of a polylysogenic *Staphylococcus aureus* strain causing the selective production of only one of its prophages. Fractionating extracts of *P*. *aeruginosa* identified the phenazine pyocyanin as a highly potent prophage inducer of *S*. *aureus* that, in contrast to mitomycin C, displayed prophage selectivity. Mutagenesis and biochemical investigations confirm the existence of a noncanonical mechanism beyond SOS-response that is controlled by the intracellular oxidation level and is prophage-selective. Our results demonstrate that human pathogens can produce metabolites triggering lysogenic to lytic conversion in a prophage-selective manner. We anticipate our discovery to be the starting point of unveiling metabolite-mediated microbe–prophage interactions and laying the foundations for a selective small molecule controlled manipulation of prophage activity. These could be for example applied to control microbial communities by their built-in destruction mechanism in a novel form of phage therapy or for the construction of small molecule-inducible switches in synthetic biology.

## Introduction

Microorganisms engage in an enormous diversity of ecological interactions that are largely controlled by small molecule metabolites.^1−3^ Chemical interactions between microbes or with their eukaryotic hosts have been extensively studied and exploited for drug discovery. However, relatively little is known about the role of one of the key players in microbial ecosystems within this network of chemical interactions: bacteriophages. This is even more surprising since bacteriophages, viruses infecting bacteria, are the most diverse and abundant biological entities on our planet.

The human body and its microbiota harbor an enormous diversity of phages. These phages drive microbial evolution and dynamically shape microbial communities.^4,5^ Altered phage composition in the gut has been linked with human diseases,^6−8^ and phages may contribute to maintenance of intestinal immune functions.^9^ The vast majority of phages in the human gut are residing integrated as prophages in the genomes of their respective microbial hosts.^10^ These prophages can be induced under certain conditions to resume a lytic lifestyle resulting in the production of virus particles (virions) and the destruction of the host cell.^11^ This typically involves inactivation of a prophage repressor via the SOS-response.^12^ Genotoxic agents like mitomycin C trigger this SOS-response through DNA damage prompting the expression of multiple genes encoding repair pathways.^13^ Although phages during the lytic cycle destroy their host, a prophage confers various fitness benefits to its microbial host.^14,15^ These include for example the introduction of toxins and other virulence related factors encoded by the prophage,^16,17^ protection from superinfection by other phages, and serotype conversion by modulating the structure of lipopolysaccharide O-antigens.^18,19^ For bacterial populations, liberated phage particles of spontaneous lysis events of individual cells also may serve as a form of bacterial warfare against nonlysogenized competitors.^14^ Thus, prophages are not only a bacterium’s Achilles heel but also important mutualistic traits and possibly even a key for controlling microbial communities.

The chemistry of microbe–phage interactions still remains underexplored, although recent work has reported defense compounds preventing lytic infections^20^ and the internal regulation of the lysis-lysogeny decision of prophages via quorum sensing signals of the bacterial host^21^ or its phages.^22,23^

Here we expand the repertoire of small-molecule-mediated microbial interactions by the discovery of a cross-species prophage inducing metabolite and prophage-selective trigger factor.

## Results

### Metabolites Cause Prophage Induction

**Figure 1:**
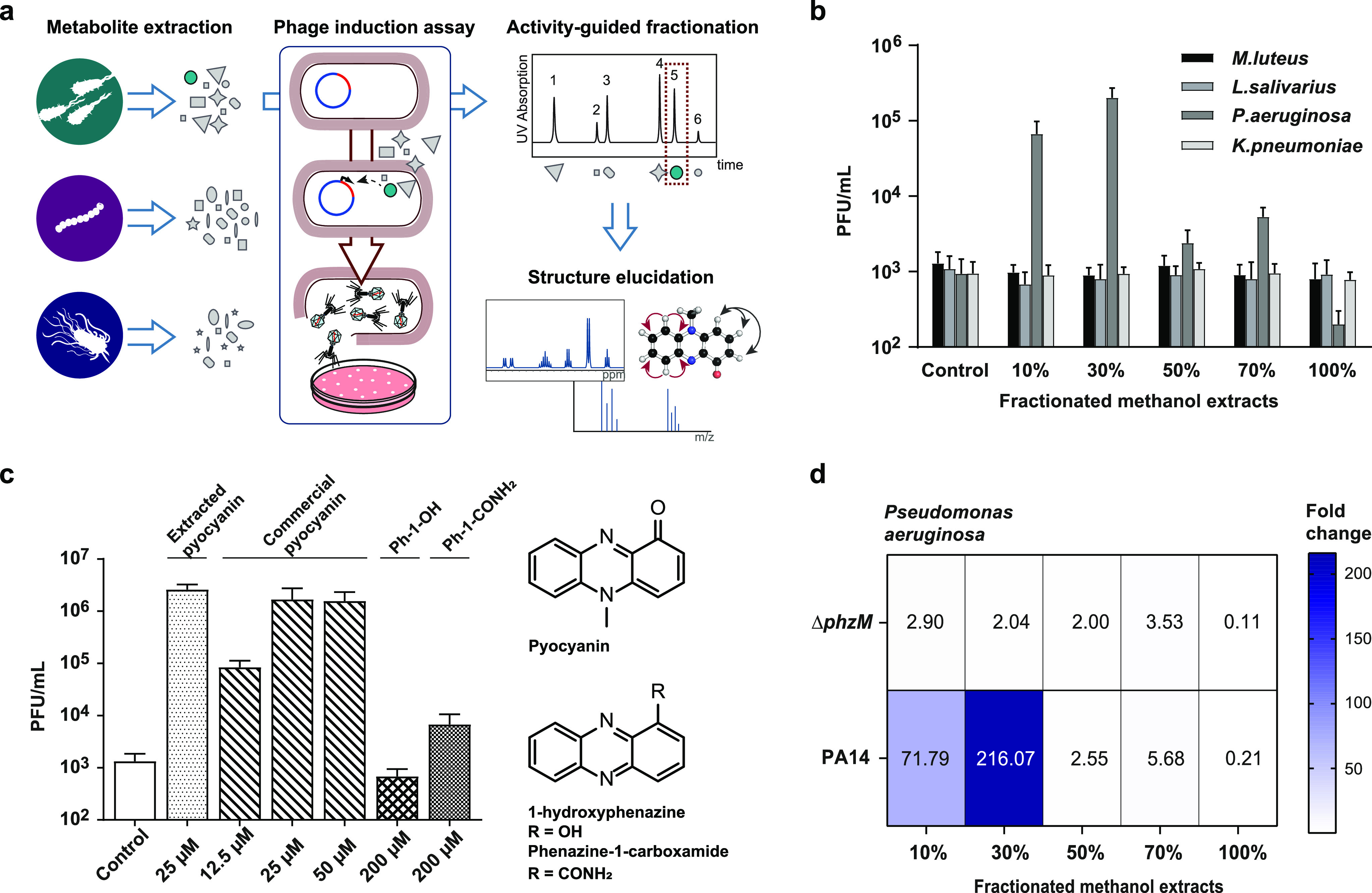
Activity-guided isolation and identification of the prophage inducer pyocyanin. (a) Screening scheme for the discovery of potential prophage inducers from microbial metabolites. (b) Two fractions of the extracts of *P*. *aeruginosa* increased phage production in *S*. *aureus* ATCC 6341. (c) The prophage inducer was identified as the phenazine pyocyanin, which compared to other phenazine compounds showed 2–3 orders of magnitude higher prophage induction. (d) Fold-induction measured by PFU counts relative to control of fractionated extracts of *P*. *aeruginosa*. In contrast to the wild type, a Δ*phz*M transposon mutant, which lacks the enzyme responsible for the N-methylation step in the pyocyanin biosynthesis, did not cause prophage induction. For parts b, c, and d three independent biological replicates were performed, and the mean PFU/mL values with the corresponding standard deviations (b, c) are reported.

We hypothesized that some microbes might exploit the vulnerable balance of the lysis–lysogeny decision by producing small molecules modulating prophage induction of their competitors. (The term prophage induction here refers to the entire process of successful production of free phage particles containing the phage genome.) To explore this possibility, we first tested the inducibility of prophages by the antibiotic mitomycin C in five human isolates of *Staphylococcus aureus* using a plaque assay. Mitomycin C is used as standard agent for phage induction,^24^ which causes DNA damage and induces phages by eliciting the bacterial SOS-response.^13^ Phage particles were quantified as plaque forming units (PFU) on agar plates with the phage-susceptible *S. aureus* reporter strain RN4220. *Staphylococcus aureus* strain ATCC 6341 gave a strong prophage induction with mitomycin C and was consequently used for a screening of solid phase extracts of culture supernatants of human commensals and pathogens that are frequently occupying the same niches (Figure 1a). While metabolite extracts from *Micrococcus luteus*, *Lactobacillus salivarius*, and *Klebsiella pneumonia* had no effects on prophage induction, extracts of *Pseudomonas aeruginosa* strikingly increased phage production by more than 2 orders of magnitude (Figure 1b). We thus aimed to isolate the prophage inducing metabolite and elucidate its structure.

### Structure Elucidation of the Inducer

The characteristic blue coloration of the active fraction suggested that the inducer may be a phenazine. The active compound was obtained by activity-guided fractionation by liquid–liquid extraction with subsequent HPLC-purification to homogeneity (Supporting Information Figure S1). High resolution mass spectrometry resulted in an *m*/*z* of 211.08635 indicating a molecular formula of C_13_H_10_N_2_O (Supporting Information Figure S2). Structure elucidation by 1D and 2D NMR spectroscopy ultimately identified pyocyanin as the active compound (Supporting Information Figure S3A–E, Table S2).

The activity of the isolated compound was identical with commercial pyocyanin and led to concentration dependent prophage induction with up to 1.7 × 10^6^ PFU/mL at 25 μM. Other phenazines of *P. aeruginosa* such as 1-hydroxyphenazine had no significant effects and phenazine-1-carboxamide only minor effects on phage production even at concentrations of up to 200 μM (Figure 1c, Supporting Information Figure S4a).

The specificity of the effect for pyocyanin was confirmed by a Δ*phz*M transposon mutant of *P. aeruginosa* which maintains an intact phenazine gene cluster but is unable to perform the N-methylation step in the biosynthesis of pyocyanin: in contrast to metabolite fractions of wild type *P. aeruginosa*, those of the Δ*phz*M mutant did not cause phage induction (Figure 1d), confirming that pyocyanin is the major phage inducer produced by *P. aeruginosa*. These results suggest that small-molecule-mediated bacterial species–species interactions indeed also involve prophage induction.

### Selectivity of Prophage Induction

Mitomycin C caused a maximum of phage induction at 1.5 μM, while at higher concentrations effective phage production was probably prevented by increasing cell toxicity. Remarkably, the effectiveness of pyocyanin in prophage induction of *S. aureus* ATCC 6341 even exceeded the maximum effect of mitomycin C by an order of magnitude (Supporting Information Figure S4b). In order to gain a more detailed understanding of prophage induction by pyocyanin, we used single-molecule real-time sequencing (PacBio) of the entire genome of *S. aureus* ATCC 6341. Analysis of the assembled genome with Phaster^25^ identified six prophage-like regions (PLR I–VI). Two of them (I and VI) were *Staphylococcus aureus* pathogenicity islands (SaPImbl1 and SaPImbl6), one (V) was tentatively characterized as incomplete prophage, and three regions (II, III, and IV) were identified as *Siphoviridae* (phiMBL2-4) (Supporting Information Figure S5). Production of phage particles was investigated by PCR analysis. We hereby took advantage of the fact that DNA packed in capsids is well protected from nucleases and can thus be differentiated from free genomic DNA of disrupted bacterial cells. The cell-free supernatants of induced cultures were DNase treated to digest genomic DNA of lysed cells, whereas DNA packed inside phage particles would remain intact (Supporting Information Figure S6a). DNase was then inactivated and the capsids were disrupted by a heat denaturation step.

**Figure 2:**
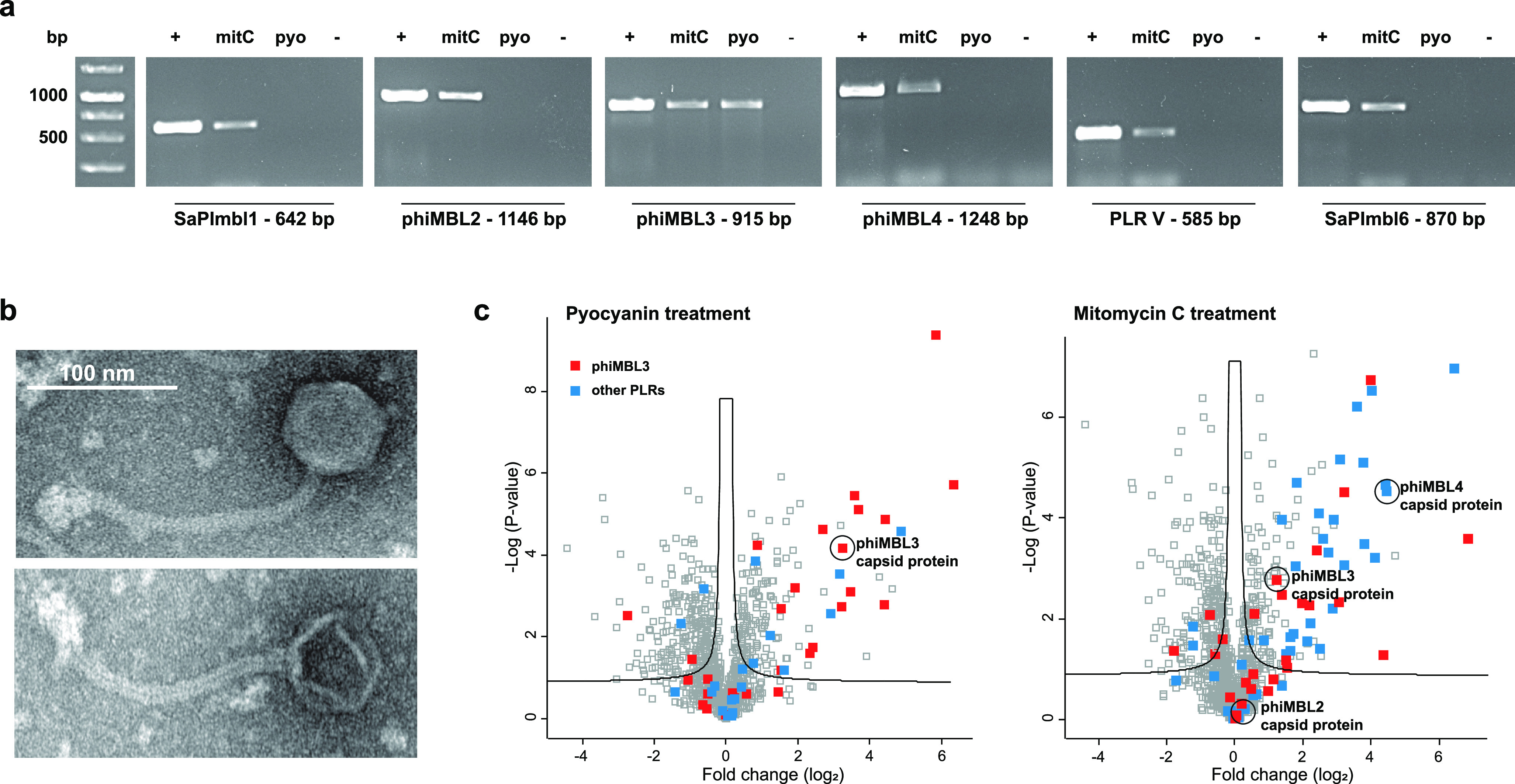
Selective prophage induction of pyocyanin in the polylysogenic *S. aureus* ATCC 6341 strain. (a) Culture supernatants of *S. aureus* ATCC 6341 cultures treated with 25 μM pyocyanin (pyo) and 1.5 μM mitomycin C (mitC). Agarose gels show amplification prophage-like regions for mitomycin C treatment, whereas pyocyanin treatment only gave successful amplification of phiMBL3. Representatives of five independent biological replicates are shown for each treatment. Isolated genomic DNA was used as positive control (+). (b) Representative TEM microscopy images of propagated and isolated phage phiMBL3. (c) Volcano plots of *p* values versus the log 2 fold change in protein abundance between treated samples and DMSO control of cell lysates of *S. aureus* ATCC 6341 cultures treated with 25 μM pyocyanin (left) and 1.5 μM mitomycin C (right) (*n* = 3; FDR 0.05; s0 0.1). While only capsid protein of phiMBL3 was detected for pyocyanin treatment, also capisd proteins of the other phages were found for mitomycin C treatment.

Subsequently, diagnostic fingerprint regions were amplified by PCR using sequence-specific primers. All of the six prophage-like regions could be detected after induction with mitomycin C, while only one (phiMBL3) was found with pyocyanin (Figure 2a, Supporting Information Figure S6b).

In order to test which of these were further infectious to phage-sensitive *S. aureus* RN4220, we added cell-free supernatants of mitomycin C and pyocyanin induced strain ATCC 6341 to a lawn of strain RN4220 on agar plates and after plaque formation collected propagated phages by PEG/NaCl precipitation. PCR analysis demonstrated for mitomycin C treated samples successful propagation of phiMBL3 and phiMBL4, while for pyocyanin treatment only phiMBL3 was propagated in the sensitive reporter strain RN4220 (Supporting Information Figure S6c).

The pyocyanin-inducible phage propagated in strain RN4220 was isolated from plaques, and next generation genome sequencing ultimately confirmed the sequence identity of phiMBL3. The induced phage exhibits partial homology with *S. aureus* phage phiJB (99.5% at 68% coverage). TEM experiments confirmed a phage with *Siphoviridae* morphology with a tail length of 180 nm and a head diameter of 60 nm (Figure 2b). Proteomic analysis of induced culture supernatants of strain ATCC 6341 showed only the presence of phiMBL3 in pyocyanin treated samples, while major capsid proteins of all three phages were detected after mitomycin C treatment (Figure 2c). These results demonstrate that pyocyanin causes prophage-selective induction of phiMBL3 in a polylysogenic *S. aureus* strain.

### Mechanism of Pyocyanin

**Figure 3:**
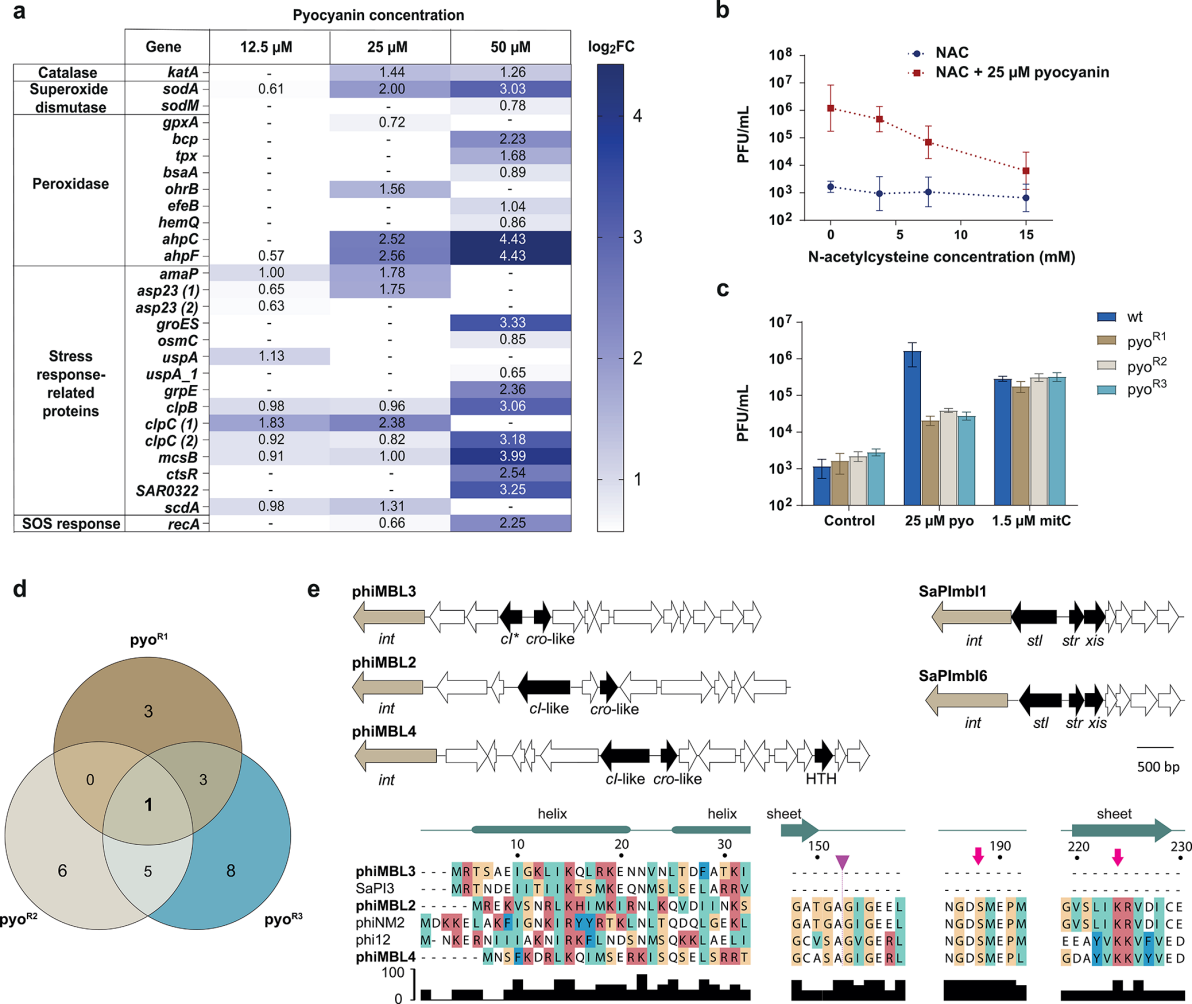
Mechanism of pyocyanin-mediated selective prophage induction. (a) Transcriptional analysis showing upregulated oxidative stress response-related genes as the log 2 fold change for three different pyocyanin concentrations in comparison to DMSO control (*n* = 3). (b) Phage production dependent on increasing concentrations of the ROS scavenger *N*-acetylcysteine (NAC) as measured by plaque formation. (c) Prophage induction by plaque formation with wild type *S. aureus* ATCC 6341 and pyocyanin resistant mutants (pyo^R1-3^) upon treatment with 25 μM pyocyanin (pyo) and 1.5 μM mitomycin C (mitC). For parts b and c three independent biological replicates were performed and the mean PFU/mL values with the corresponding standard deviations are shown. (d) Venn diagram of mutations shared between the three independently generated pyocyanin mutants. (e) Genome maps of the lytic–lysogenic genes of the prophages and pathogenicity islands in *S. aureus* ATCC 6341. Phage phiMBL3 harbors a truncated CI*-like repressor which lacks the C-terminal domain with cleavage site (purple triangle) and active site (pink arrows).

Differential gene expression of pyocyanin treated *S. aureus* ATCC 6341 in comparison to DMSO controls revealed upregulated expression levels of different phage genes but also multiple oxidative stress response genes, pointing to the mechanism of pyocyanin (Figure 3a, Supporting Information Table S3). Pyocyanin is known to interfere with the electron transport chain in *S. aureus* and to cause oxidative stress.^26,27^ Using a 2′,7′-dichlorofluorescein diacetate (DCF-DA) fluorescent sensor for reactive oxygen species (ROS), we confirmed a strong increase of ROS in *S. aureus* ATCC 6341 upon treatment with pyocyanin at concentrations used for prophage induction (Supporting Information Figure S7a). We thus investigated if scavengers of reactive oxygen species would protect cells from pyocyanin-mediated prophage induction. Indeed, *N*-acetylcysteine significantly reduced the production of the phage in concentration dependence by more than 2 orders of magnitude, suggesting that the oxidative environment rather than any direct interaction of pyocyanin with a host protein is responsible for prophage induction (Figure 3b).

To investigate whether any type of ROS or electron transport chain inhibition would cause phage induction at levels and selectivity comparable to pyocyanin, we tested hydrogen peroxide and *trans*-Δ^1^-NQNO, a highly effective antistaphylococcal metabolite of *P. aeruginosa*.^28^ In wild type *S. aureus* ATCC 6341 even concentrations as high as 1 mM of hydrogen peroxide only led to a 2-fold increased phage production, which is roughly 3 orders of magnitude below induction by pyocyanin. Also the quinolone *N*-oxide, which has been shown to interfere with the *S. aureus* electron transport chain,^28^ only caused a maximum phage induction of 1 order of magnitude (Supporting Information Figure S7b).

This low responsiveness matched considerably lower ROS levels that were measured for hydrogen peroxide and *trans*-Δ^1^-NQNO treatment compared to pyocyanin (Supporting Information Figure S7a). More importantly, while pyocyanin was selective for phage phiMBL3, both hydrogen peroxide and *trans*-Δ^1^-NQNO led to production of multiple phages (Supporting Information Figure S7c). Similar to mitomycin C, also hydrogen peroxide is known to elicit the SOS-response in *S. aureus* by oxidative DNA damage^29^ and quinolone *N*-oxides may indirectly cause the same effect.^30^ These results suggest that the mechanism of induction by pyocyanin relies on a high level of ROS production but strictly differs from that of other oxidants and electron transport chain inhibitors.

With the aim to gain further insights into the mechanism and its connection to prophage induction, we selected three pyocyanin resistant (pyo^R^) mutants of *S. aureus* ATCC 6341 over several days of continuous exposure. These pyo^R^ mutants exhibited MIC values for pyocyanin of 150–200 μM corresponding to up to 4-fold enhanced tolerance compared to the wild type. In comparison to the wild type, pyo^R^ mutants displayed identical phage induction with mitomycin C but approximately 2 orders of magnitude lower response to pyocyanin, confirming the different induction mechanisms of both compounds (Figure 3c). Next generation sequencing of the pyo^R^ mutants revealed mutations (deletion and nonsense) in the NAD(P)/FAD-dependent oxidoreductase gene as the only shared feature (Figure 3d, Supporting Information Table S4). Mutation of the oxidoreductase likely limited redox cycling of pyocyanin and thereby decreased oxidative stress and hence prophage induction. We suspected that the mechanistic differences for the induction of the prophages may be reflected by different types of prophage repressors.

Comparing the sequences of all prophage-like elements in the genome of *S. aureus* ATCC 6341 revealed major differences between the three prophages (Supporting Information Figure S8a). While phiMBL2 and phiMBL4 use the standard CI-like repressor, which is typically inactivated by SOS-response mediated RecA-dependent autoproteolytic processing, phiMBL3 exhibits a C-terminally truncated CI*-like repressor lacking the protease domain (Figure 3e, Supporting Information Figure S8b). Consequently, the phiMBL3 repressor cannot be derepressed by RecA-mediated self-cleavage. Consistent with these findings, a phylogenetic analysis revealed that the CI*-like repressor of phiMBL3 clustered with Stl repressors of pathogenicity islands (SaPIs) which are typically derepressed by other proteins instead of being cleaved (Supporting Information Figure S8c,d).

### Induction Selectivity and Cell Viability

The selective induction of *S. aureus* phage phiMBL3 by pyocyanin raised the question of how its producer *P. aeruginosa* might benefit from this selectivity. We speculated that a broad spectrum SOS-response inducing compound would also induce resident prophages in the genome of *P. aeruginosa* which would be self-destructive.

**Figure 4:**
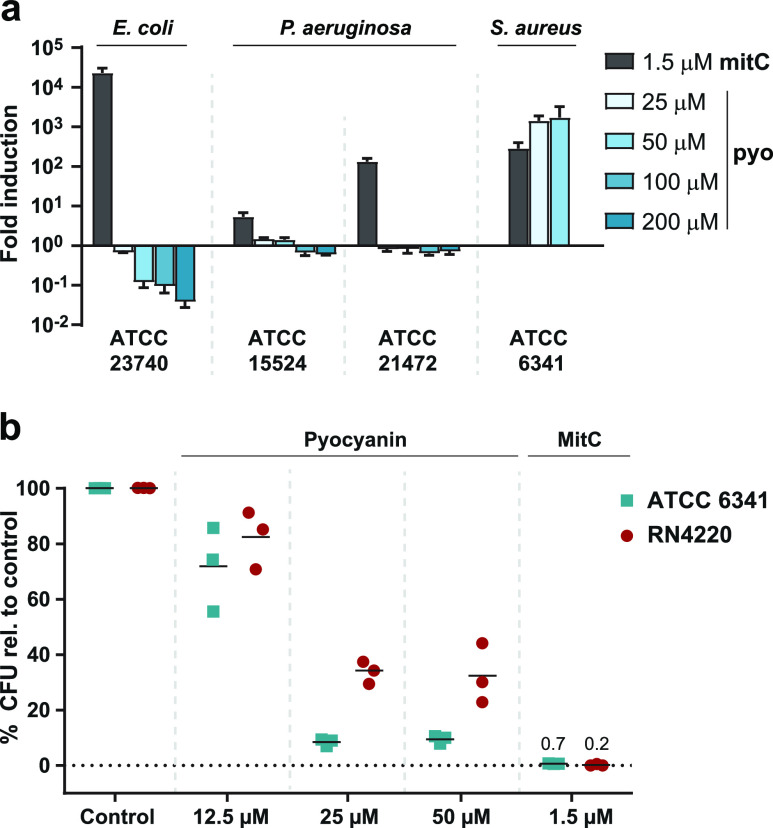
Species-selectivity of prophage induction. (a) Prophage induction relative to controls (fold induction) measured by plaque forming units for *E. coli* and *P. aeruginosa* strains upon treatment with different pyocyanin (pyo) concentrations and mitomycin C (mitC) using corresponding phage-sensitive indicator strains. (b) Percentage of colony forming units (CFU) of *S. aureus* ATCC 6341 and prophage-cured *S. aureus* RN4220 relative to DMSO control after treatment with different pyocyanin concentrations and mitomycin C. For each compound and concentration, three biological assay replicates were performed.

We thus compared the activity of pyocyanin and mitomycin C for several prophage-harboring strains of *P. aeruginosa*. Indeed, while mitomycin C caused prophage induction, pyocyanin had no such effect (Figure 4a). We additionally aimed to assess if this immunity to the effects of pyocyanin was specific to *P. aeruginosa* or a more general trait among Gram-negative bacteria. We thus additionally tested *Escherichia coli* strain ATCC 23740 which was sensitive to growth inhibition by pyocyanin at high concentrations. While mitomycin C induced a lambdoid phage in *E. coli* ATCC 23740 that was identified by genome sequencing (99.96% sequence identity to phage λ which is deposited under NC_001416.1), pyocyanin did not lead to induction even at concentrations up to the MIC value of 200 μM and in contrast even slightly reduced PFU counts in concentration dependence (Figure 4a). These results show that pyocyanin also exhibits selectivity on a species level and does not induce prophages of *P. aeruginosa* or *E. coli*. We next aimed to explore if induction of prophages significantly contributes to cell lysis. We thus compared the colony forming units (CFU) of *S. aureus* ATCC 6341 with the prophage-cured strain RN4220 upon pyocyanin treatment. Strain ATCC 6341 showed significantly reduced viability at prophage-inducing pyocynanin concentrations compared to RN4220. These results suggest that prophage induction may actively contribute to killing of cells probably due to phage-related cell disruption and indicate that prophage induction may be a suitable strategy for *P*. *aeruginosa* to control population density of competing bacteria (Figure 4b).

## Discussion

We have demonstrated that pyocyanin produced by *Pseudomonas aeruginosa* triggers the lysogenic to lytic conversion of a polylysogenic *Staphylococcus aureus* strain, and it does so in an unprecedented prophage-selective manner only leading to the production of phage phiMBL3. *Pseudomonas aeruginosa* and *Staphylococcus aureus* frequently cocolonize in the lungs of cystic fibrosis patients, and pyocyanin concentrations of up to 100 μM have been detected in sputum samples.^31,32^ This suggests that prophage-inducing pyocyanin concentrations may be realistically achieved in the human body. Consistent with our findings, a previous study has shown that several prophage genes were upregulated in transcription profiles of *S*. *aureus* grown in coculture with *P. aeruginosa*.^33^

Prophage induction mediated by the SOS-response has been shown, for example, for *S*. *aureus* and *Escherichia coli* that were exposed to H_2_O_2_, β-lactam antibiotics, or ciprofloxacin.^34−37^ However, the efficiency of prophage induction was typically much less than that of pyocyanin and no selectivity at the prophage level was observed. While pyocyanin was prophage-selective, mitomycin C and other SOS-response causing agents induced several prophages and pathogenicity islands (SaPIs). The mechanism responsible for the selectivity of pyocyanin consequently operates independently from the SOS-response. Pyocyanin-mediated prophage induction depends on high-level production of reactive oxygen species and is controlled by the intracellular oxidation level. The truncated CI*-like repressor of phiMBL3 resembles Stl-repressors of SaPIs, which are known to be induced by multiple diverse and unrelated proteins of helper phages. It can thus be speculated that the repressor of phiMBL3 may be specifically derepressed in an oxidative cellular environment by binding of an oxidative stress or redox state-induced or -activated protein which still needs to be discovered. In *E. coli* oxidative stress is known to activate the transcription factor OxyR, which efficiently represses prophage induction.^38^ This effect may be responsible for the nonresponsiveness of Gram-negative bacteria to pyocyanin. In addition, some Gram-negative bacteria have potent detoxification mechanisms such as efflux pumps that can reduce effective exposure to pyocyanin.^39^ In a sense, species selectivity possibly entails prophage selectivity, and since most bacteria harbor prophages, secreting metabolites with nonselective activity would backfire on their producers. *P. aeruginosa* may benefit from lysis of a subpopulation of a particular *S. aureus* strain but also from the cascade effect that phage production may have on other nonlysogenized competitors. Phage particles may even trigger a maladaptive antiviral immune response that prevents clearance of a bacterial pathogen and aid the infection process in mammalian hosts.^40^ Pyocyanin has many important biological functions including mediating cell death of *P*. *aeruginosa* during biofilm formation, promoting anaerobic survival, and acting as virulence factor against the human host.^41−43^ Our finding that pyocyanin also serves as a selective prophage inducer adds to the long list of remarkable activities of this metabolite.

Recent discoveries have increasingly contributed to a picture of small molecules driving microbe–phage interactions within a species. For example, a quorum sensing signal was identified that guides the lysogenic to lytic decision of *Vibrio* depending on cell density,^21^ and it was found that a peptide-based signal called arbitrium enables communication between lysogenic phages.^23^ Also *Streptomyces* metabolites have been described acting as a chemical defense system to block replication of lytic phages via intercalating DNA.^20^

We now demonstrated that microbial metabolites can be potent cross-species prophage inducers and furthermore exhibit selectivity on a prophage level in polylysogenic hosts. Our results not only challenge the understanding of metabolite-mediated interactions within microbiota by adding an additional layer of complexity but also open up an entirely new field for manipulating these interactions. Selective prophage inducers could allow exploitation of a built-in destruction mechanism of microbes leading to controlled lysis. Triggering lysogenic to lytic conversion in a selective way may allow active restructuring of microbial communities and may offer an alternative solution to phage therapy. On the other hand, it also has not escaped our notice that the selective induction of prophage-like sequences could be repurposed for the construction of small molecule controlled molecular switches for synthetic biology.

## Conclusion

We have shown that pyocyanin produced by *Pseudomonas aeruginosa* is a trigger factor that hijacks the lysogenic to lytic switch of a polylysogenic *Staphylococcus aureus* strain and leads to the selective production of only one of its prophages. We provide evidence that this potent and prophage-selective induction is controlled by a mechanism that differs from the typical SOS-response.

## Supporting Information Available

Microbial strains, NMR data, RNA-seq results, mutant sequencing data, mass spectra, proteomics data, genome analyses, ROS detection, phage quantification, and PCR experiments (PDF)
