# Supplementary material for: A Metabolite of Pseudomonas Triggers Prophage-Selective Lysogenic to Lytic Conversion in Staphylococcus aureus
Source: J Am Chem Soc. 2021 May 12;143(22):8344–51. doi: 10.1021/jacs.1c01275 (PMC8193634; doi:10.1021/jacs.1c01275)
Supplement: Supplementary file 1 — ja1c01275_si_001.pdf [file ja1c01275_si_001.pdf]

A Metabolite of Pseudomonas triggers Prophage-selective Lysogenic to Lytic Conversion in *Staphylococcus aureus*

Magdalena Jancheva and Thomas Böttcher*

Department of Chemistry, Konstanz Research School Chemical Biology, Zukunftskolleg, University of Konstanz, 78457 Konstanz, Germany
Faculty of Chemistry, Department of Biological Chemistry & Centre for Microbiology and Environmental Systems Science, University of Vienna, 1090 Vienna, Austria

Materials and Methods

1. <u>Chemicals</u>

All solvents used in the study were purchased from Roth, Sigma-Aldrich or VWR Chemicals. Pyocyanin, mitomycin C, 2',7'-dichlorofluorescin diacetate and *N*-acetylcysteine were from Sigma-Aldrich, 1-hydrohyphenazine from TCI Chemicals, phenazine-1-carboxamide from Santa Cruz Biotechnology.

2. <u>Bacterial strains and extractions</u>

The bacterial strains used in this study are listed in Table S1. In all experiments, *Staphylococcus aureus* strains were grown in CCY medium (yeast extract 30 g/L, casamino acids 20 g/L, sodium pyruvate 20 g/L, 2.5 g NaH_2PO_4, 0.42 g/L KH_2PO_4) or LB medium (Roth). *Escherichia coli*, *Pseudomonas aeruginosa* and *Klebsiella pneumonia* were grown in LB Broth (Roth), *Micrococcus luteus* in NBE medium (meat extract 1 g/L, yeast extract 2 g/L, casein peptone 5 g/L, NaCl 5 g/L) and *Lactobacillus salivarius* in MRS Broth (Sigma).
For the secondary metabolite extraction, overnight bacterial cultures were diluted 1:100 in the corresponding medium. *P. aeruginosa* and *K. pneumoniae* were incubated for 48 h at 37˚C, *L. salivarius* for 72 h at 37˚C and *M. luteus* for 5 days at 30˚C with shaking. After the incubation period, the 200 mL cultures were centrifuged, and the supernatant was sterile filtered through 0.2 µm Filtropur BT25 bottle top filters (Sarstedt). The extracts were lyophilized, loaded on Waters C18 Sep-Pak® vac 12cc columns and eluted with different methanol concentrations (10-100%). For the phage induction assay, the dried fractions were re-constituted in 500 µL DMSO.

3. <u>Pyocyanin isolation and purification</u>

Pyocyanin extraction was carried out as described previously (*1*). Briefly, 500 mL of sterile filtered culture supernatant of PA14 were extracted with chloroform (1:2). The chloroform layer was then re-extracted with 0.2 M HCl until a colour change was observed (blue to red). To the aqueous HCl layer, Na-borate buffer (pH 10) was added until the colour turned blue again. In the last step, the pyocyanin was re-extracted in chloroform.
The extracted pyocyanin was further purified by preparative HPLC with a Reprosil-Pur Phenyl 10 µm, 250 x 20 mm column (Dr. Maisch, Ammerbuck, Germany). The flow rate was 15 mL/min. Eluent A was 0.1% formic acid in water and eluent B was 0.1% formic acid in methanol. The gradient elution was for 15 min 10% eluent B followed by 25 min 10-95% eluent B. NMR spectra

of pyocyanin was obtained with Bruker Avance Neo spectrometer at 800.30 MHz frequency (1H) and a frequency of 201.25 MHz (13C). Chemical shifts (δ) are given in parts per million (ppm) relative to the solvent residual signal of MeOD-d_4 = 3.31 ppm. The measured data was processed and analysed with MestreNova 12.0.4 software. Mass spectrometry data were obtained on an ESI-Orbitrap (Thermo Scientific, LTQ Orbitrap Velos) by direct injection and analyzed with Xcalibur (Thermo Scientific) software.

4. Phage induction and propagation

Overnight cultures of phage host strains were prepared from a glycerol cryo stocks in 5 mL LB. After overnight incubation at 37˚C, 180 rpm, the cultures were diluted 1:100. At exponential phase (OD_{600}=0.8), the culture was split in parts and 10 µL bacterial extracts (in DMSO) or different concentrations of the commercial compounds were added in a final volume of 2 mL. The culture was further incubated at 37˚C, 180 rpm. After 4 h, 1 mL was centrifuged at 3000 rpm at 4˚C for 10 min. The supernatant was collected, sterile filtered through 0.2 µm pore diameter membrane filter and stored at 4˚C until use.
For the *S. aureus* plaque assays, 100 µL of the phage supernatant and 300 µL of an overnight culture of the indicator strain RN4220 were mixed in a glass tube containing 3 mL top agar (0.6% agar) and poured onto agar plates supplemented with 10 mM $CaCl_2$. The plates were incubated overnight at 37˚C to form plaques on the lawns. In the plaque assay experiments with *E. coli* and *P. aeruginosa*, same procedure was applied except for using 100 µL of the indicator strains *E. coli* 3925 and *P. aeruginosa* PA14.

5. MIC assay and colony forming units (CFU) counts

From overnight culture of the different strains and the generated mutants, 1:1000 dilution in LB and CCY medium were made. In a 96-well plate, 1 µL of pyocyanin diluted in DMSO with 99 µL of the diluted bacteria were added. The plate was incubated at 37°C at 180 rpm. The plates were read out the next day. An inhibition was considered positive if no haze of the medium and no cell pellet was visible. MIC values were determined in triplicates.
S. aureus ATCC 6341 and RN4220 samples treated with different pyocyanin concentrations were diluted in CCY medium and 10 µL of the corresponding dilutions were spread on LB agar plates. The plates were then incubated overnight at 37˚C and the next day CFU were counted for each. Results are expressed as survival percentage from a non-treated DMSO control.

6. Phage precipitation, DNA extraction and TEM microscopy

5 mL of SM buffer (50 mM Tris-HCl, pH 7.5, 100 mM NaCl, 10 mM $MgSO_4$, 0.01% gelatin) were added to plates with developed plaques and the plates were kept at 4˚C overnight. The next day, 4 mL of phage solution was collected from the plates, centrifuged at 10.000 rpm for 10 min and sterile filtered. To the supernatant, an equal amount of 20% PEG 8000/2.5 M NaCl solution was added. Afterwards, phages were allowed to precipitate on ice for 30 min. The phage solution was then centrifuged at 10.000 rpm for 10 min, the supernatant was discarded, and the precipitate dissolved in 2 mL TMN buffer (10 mM Tris-HCl, pH 7.5, 50 mM NaCl, 5 mM $MgCl_2$). DNase and RNase were added in a final concentration of 2.5 µg/mL and the phage solution was incubated for 30 min at 37˚C. The PEG/NaCl precipitation procedure was repeated once more and the supernatant was thoughtfully discarded by two consecutive centrifugations at 10.000 rpm for 10 min and 5.000 rpm for 2 min, respectively. The visible precipitated phage pellet was collected with

200 µL TMN buffer or sterile H_2O (for TEM experiments) and the solution was stored at 4˚C until use (*2*).
The phage DNA was extracted using the Phage DNA Isolation Kit (Novagen, Canada). The NGS sequencing was performed by Eurofins GATC Biotech GmbH (Konstanz, Germany). The obtained sequence was searched for homology in the NCBI database using BlastN, BlastP and ORF finder (http://www.ncbi.nlm.nih.gov).
For the TEM microscopy experiment on Zeiss EM 912 Omega (Carl Zeiss AG, Oberkochen, Germany), 10 µL of the precipitated phage solutions fixed with 0.5% glutardialdehyde were negatively strained with 2% uranyl acetate and loaded on carbon-coated copper grids. The images were taken with TRS slow scan CCD-camera for TEM (Tröndle Restlichtverstärker Systeme, Moorenweis, Germany) at 20,000-80,000-fold magnification.

7. *De novo* DNA sequencing and Differential Gene Expression experiments

S. aureus ATCC 6341 and *E. coli* ATCC 23740 were purchased from the German Collection of Microorganisms and Cell Cultures (DSMZ). DNA from these strains was extracted with the Invitrogen™ PureLink™ Microbiome DNA purification kit as recommended by the manufacturer (ThermoFisher Scientific). The PacBio SMRT sequencing, library preparation, *de novo* assembly and annotation for *S. aureus* ATCC 6341 was carried out by Microsynth AG (Balgach, Switzerland). *E. coli* ATCC 23740 was sequenced at Eurofins GATC Biotech GmbH (Konstanz, Germany). Sequenced genomes of both strains were deposited at DDBJ/ENA/GenBank. The genome of *S. aureus* ATCC 6341 was deposited under BioProject ID PRJNA726939, BioSample accession SAMN18981021. The genome of *E. coli* ATCC 23740 under BioProject ID PRJNA727601, BioSample accession SAMN19026093.
For the NGS RNA-sequencing experiment, *S. aureus* ATCC 6341 was grown to OD_{600} 0.8 in LB medium and was then treated with 3 different pyocyanin concentrations (12.5, 25 and 50 µM) with DMSO as control. After 3.5 h of incubation at 37˚C 180 rpm, 1 mL of the cells were pelleted and transferred to DNA/RNA Shield™ Lysis Tubes (Zymo Research). Cells were lysed with FastPrep-24™ 5G instrument (MP Biomedicals) at 6.0 m/s for 40 s. The lysis cycle was repeated twice with a 300 sec break on ice. RNA isolation, Illumina Next-Seq (10 million reads 1*75) and the differential gene expression analysis was performed at Microsynth AG (Balgach, Switzerland) with using *S. aureus* ATCC 6341 as a reference strain.

8. Bioinformatics analysis

Protein homologies were identified by BlastP (https://blast.ncbi.nlm.nih.gov/Blast.cgi?). Dotplots of phage genomes were generated with the program Genome Pair Rapid Dotter, Gepard 1.40 (http://cube.univie.ac.at/gepard) (*3*) using a word length of 9 and a window size of 0. Sequence alignments were performed with PRALINE (*4*) with the color schemes adapted from CLUSTALX. Secondary structures were predicted in PRALINE using DSSP (*5*) and PSIPRED (*6*). Phylogenetic analyses were performed by generating maximum likelihood trees with bootstrap test (500 replications) using MEGA-X.

9. Proteomic analysis

The samples from the different treatments with 25 µM pyocyanin, 1.5 µM mitomycin C and DMSO as control in CCY medium were lysed with FastPrep-24TM 5G instrument (MP Biomedicals) at 6.0 m/s for 40 s. The lysis cycle was repeated twice with a 300 sec break on ice.

All samples were reduced with DTT (30 min, 56°C) and alkylated with chloroacetamide (60 min, RT). Digestions were performed using trypsin (16 h, 30°C). The digests were analysed on a QExactive HF mass spectrometer (ThermoFisher Scientific, Bremen, Germany) interfaced with an Easy-nLC 1200 nanoflow liquid chromatography system (ThermoFisher Scientific, Bremen, Germany). The peptide digests were reconstituted in 0.1 % formic acid and loaded onto the analytical column (75 µm × 15 cm). Peptides were resolved at a flow rate of 300 nL/min using a linear gradient of 5−32% solvent B (0.1% formic acid in acetonitrile) over 45 min. Data-dependent acquisition with full scans in a 350 − 1500 m/z range was carried out at a mass resolution of 120000. The 10 most intense precursor ions were selected for fragmentation. Peptides with charge states 2−7 were selected, and dynamic exclusion was set to 30 sec. Precursor ions were fragmented using higher-energy collision dissociation (HCD) set to 28%. Tandem mass spectra were searched against a suitable protein database using Mascot (Matrix Science) with "Trypsin/P" enzyme cleavage, static cysteine alkylation by chloroacetamide and variable methionine oxidation.

10. PCR sample preparation and setup

For this experiment, the steps of phage induction and propagation were conducted as described previously, but instead of CCY-Medium, LB-Medium was used to minimize the background of the control. The web-based software Phaster (*7, 8*) was used to map the prophage-like regions (PLRs) in the genome of *S. aureus* ATCC 6341. For the complete phages (phiMBL2, phiMBL3 and phiMBL4) the major capsid protein genes were selected for amplification, whereas for the pathogenicity islands (SaPImbl1 and SaPImbl6), corresponding SaPI-specific genes were chosen. For PLR V which we tentatively classified as incomplete prophage, a phage scaffold capsid protein gene was amplified. The primers that were designed for each of the PLR genes were synthesized by Metabion AG (Munich, Germany). Genomic DNA was used as positive control and non-induced samples (DMSO) served correspondingly as negative controls in the assay. For each treatment five biological replicates were performed.

Initially, 100 µL of the phage supernatant was transferred into a new tube, 0.1 µL of DNase (10 mg/mL) was added and incubated for 15 min at room temperature. In order to inactivate the DNase, the sample was incubated for 5 min at 75°C. The PCR reaction tubes contained: 7 µL milli-Q H_2O, 1 µL sample or genomic DNA, 1 µL forward primer, 1 µL reverse primer (final concentration 250 ng/mL) and 10 µL Phusion high-fidelity PCR master mix (ThermoFisher Scientific). The setup for the PCR reaction was as follows:

98°C (30 sec) → 35·[98°C (5 sec) → T_m-2°C (12 sec) → 72°C (20 sec)] → 72°C (10 min) → 7°C (store).

11. Gel electrophoresis

Agarose gels for electrophoresis were prepared by heating 0.5 g of agarose in 50 mL 1x TAE buffer (Roth). After cooling, 2.5 µL of peqGREEN (Peqlab, VWR) were added to the gels and mixed well before solidification. On each of the gels, 1 µL of peqGOLD 1 kb DNA ladder (Peqlab, VWR) and 5 µL of the PCR amplicon mixed with 1 µL 6xLoading dye (Peqlab,VWR) were loaded. The gels were run for 50 min at 70V with EasycastTMB1A (ThermoFisher Scientific) electrophoresis system and visualized with the Fusion-FX7 Advanced of Vilber Lourmat (Eberhardzell, Germany).

12. Reactive oxygen species (ROS) quantification

The production of ROS upon treatment with pyocyanin was quantified using the cell permeable fluorogenic dye 2',7'-dichlorofluorescin diacetate (DCFDA). The bacterial cells were grown to OD_{600} 0.8, washed with PBS and stained with 20 µM DCFDA for 30 min 37˚C with shaking. After the incubation, the cells were again washed with PBS and re-suspended in the corresponding medium. 99 µL were added to black Corning 96-well plates containing different concentrations of pyocyanin, *trans*-Δ^1-NQNO or 1 mM H_2O_2 in a final volume of 100 µL. The plate was incubated for 2 h at 37˚C with shaking (180 rpm) and fluorescence was subsequently measured on a Tecan microplate reader with Ex/Em=485/535 nm.

13. <u>ROS scavenging assay</u>

N-acetylcysteine (NAC) was used as ROS scavenger in the assay at different concentrations (3.75, 7.5 and 15 mM). As in most experiments, cells were grown to OD_{600} 0.8 in CCY medium and treated with 25 µM pyocyanin in combination with NAC. The NAC alone served as negative control in each of the indicated concentrations. After incubation and centrifugation, a plaque assay was performed for phage quantification.

14. <u>Mutant generation</u>

Overnight culture of *S. aureus* ATCC 6341 was diluted 1:10k and spread on an LB agar plate. The next day, one colony from the plate was picked and added to 3 mL of fresh LB. After reaching stationary phase, 0.3 µL of the grown culture were added to 3 mL LB tubes containing 12.5, 25, 50 and 100 µM pyocyanin and incubated overnight at 37˚C 180 rpm. From the tube with the highest concentration where bacterial growth was observed a 1:10k dilution was made in fresh LB medium tubes containing the same pyocyanin concentration. This procedure was repeated every day for 2 weeks. Afterwards, the bacteria which grew in the tube with the highest pyocyanin concentration were pelleted by centrifugation, resuspended in PBS and spread on LB plate with 100 µM pyocyanin (two times the MIC). The plate was incubated for 3 days on 37˚C. The colonies that grew after the incubation period were resuspended in 3 mL LB containing 50 µM pyocyanin (one time the MIC) (*9*). The next day, cryostocks in 15% glycerol were prepared and the MIC value for each of the mutants was determined prior to sequencing. The experiment was performed in three independent biological replicates. Genomic DNA of the mutants was extracted with the Invitrogen™ PureLink™ Microbiome DNA purification kit (ThermoFisher Scientific). The Illumina library preparation, sequencing and mutant analysis was performed at Microsynth AG (Balgach, Switzerland). The gene mutations that were common to all mutants were PCR amplified and confirmed by Sanger sequencing at Eurofins GATC Biotech GmbH (Konstanz, Germany).

Supporting Tables and Figures

Table S1.
Bacterial strains used in the study.

Species	Strain	Description	Source
Escherichia coli	ATCC 23740	λ phage host	DSMZ (DSM 8589)
Escherichia coli	DSM 3925	λ indicator strain	DSMZ
Klebsiella pneumoniae	DSM 681		DSMZ
Lactobacillus salivarius	DSM 20492		DSMZ
Micrococcus luteus	ATCC 4698		ATCC
Pseudomonas aeruginosa	PA14		DSMZ (DSM 19882)
Pseudomonas aeruginosa	PAO1 Δ*phzM* (PW8141)	phenazine-1-carboxylate *N*-methyltransferase mutant	Manoil Lab two-allele library No. PA4209
Pseudomonas aeruginosa	ATCC 15524	prophage host	ATCC
Pseudomonas aeruginosa	ATCC 21472	prophage host	ATCC
Staphylococcus aureus	ATCC 6341	*pvl*$^+$, human finger isolate, VI prophage regions	DSMZ (DSM 11110)
Staphylococcus aureus	RN4220	indicator strain	DSMZ (DSM 26309)

Table S2.
Pyocyanin NMR assignment data.

Position	δ ^{1}H (multip., *J* (Hz), no. H)	δ ^{13}C
**1**		178.31
**2**	6.60 (d, 8.7, 1H)	115.71
**3**	7.91 (t, 8.0, 1H)	146.91
**4**	6.53 (d, 7.7, 1H)	94.67
**4a**		136.63
**5-CH_3**	4.21 (s, 3H)	36.24
**5a**		134.65
**6**	8.33 (d, 7.3, 1H)	134.09
**7**	7.71 (t, 7.3, 1H)	127.42
**8**	8.03 (t, 7.5, 1H)	137.59
**9**	8.08 (d, 8.7, 1H)	116.71
**9a**		138.37
**10a**		147.16

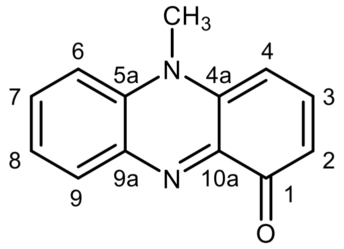

MeOD-d_4: **^{1}H NMR (800 MHz)**

^{13}C NMR (201 MHz)

HRMS **m/z calculated for $C_{13}H_{10}N_2O$ [M+H]$^{+}$: 211.08659, found 211.08635**

Table S3.
Upregulated transcription of genes (RNA-Seq) in prophage-like regions of *S. aureus* ATCC 6341 following treatment by 25 µM pyocyanin given as the log2 fold change (log_2FC) of treated sample vs. DMSO control ($p<0.05$; n=3).

Prophage-like region	Gene description	log_2FC
SaPImbl1	site-specific integrase	0.84
SaPImbl1	XRE transcriptional regulator	2.80
SaPImbl1	helix-turn-helix domain-containing protein	2.70
SaPImbl1	hypothetical pathogenicity island protein	1.48
SaPImbl1	hypothetical protein	2.18
SaPImbl1	hypothetical protein	2.48
SaPImbl1	DUF1474 family protein	3.37
SaPImbl1	DNA primase	3.14
SaPImbl1	hypothetical protein	2.17
SaPImbl1	pathogenicity island protein	1.93
SaPImbl1	pathogenicity island protein	1.64
SaPImbl1	pathogenicity island protein	1.91
SaPImbl1	hypothetical mobile element-associated protein	2.03
SaPImbl1	pathogenicity island protein	1.79
SaPImbl1	spore coat protein	2.07
SaPImbl1	pathogenicity island protein	1.48
SaPImbl1	terminase small subunit	1.83
phiMBL2	site-specific integrase	1.49
phiMBL2	hypothetical protein	0.73
phiMBL2	toxin-antitoxin system, antitoxin component	1.54
phiMBL2	hypothetical protein	0.67
phiMBL2	antirepressor	2.28
phiMBL2	hypothetical protein	1.18
phiMBL2	hypothetical protein	3.28
phiMBL2	hypothetical protein	1.29
phiMBL2	RecF/RecN/SMC N terminal domain protein	2.21
phiMBL2	recombinase	3.15
phiMBL2	MBL fold metallo-hydrolase/beta-lactamase	1.78
phiMBL2	DnaD domain protein	2.32
phiMBL2	PVL ORF-50-like family phage protein	4.55
phiMBL2	hypothetical protein	2.66
phiMBL2	hypothetical protein	5.14
phiMBL2	transcriptional regulator/RinA family	1.41
phiMBL2	phage major capsid protein	1.32
phiMBL2	putative holin-like toxin	0.83
phiMBL2	chemotaxis-inhibiting protein CHIPS	0.71
phiMBL2	hypothetical protein	0.80
phiMBL3	site-specific integrase	0.77
phiMBL3	XRE transcriptional regulator	1.56
phiMBL3	hypothetical protein	2.87
phiMBL3	hypothetical protein	1.87

phiMBL3	hypothetical protein	0.78
phiMBL3	antirepressor	2.43
phiMBL3	hypothetical protein	2.77
phiMBL3	DUF1270 family protein	2.11
phiMBL3	XRE transcriptional regulator	1.54
phiMBL3	DUF1108 family protein	4.07
phiMBL3	siphovirus Gp157 family protein	1.14
phiMBL3	phage nucleotide-binding protein	2.40
phiMBL3	phi PV83 orf 19-like protein	2.12
phiMBL3	replication protein	2.09
phiMBL3	AAA family ATPase	1.61
phiMBL3	phage N-6-adenine-methyltransferase	2.73
phiMBL3	phage phi PVL ORF 52-like protein	2.75
phiMBL3	dUTPase	2.26
phiMBL3	DUF1381 domain-containing protein	3.95
phiMBL3	hypothetical protein	1.26
phiMBL3	PBSX family phage terminase large subunit	0.98
phiMBL3	phage portal protein	0.66
phiMBL3	DUF4355 domain-containing protein	2.48
phiMBL3	phage major capsid protein	3.10
phiMBL3	DUF3168 domain-containing protein	1.30
phiMBL3	phage tail protein	2.51
phiMBL3	phage baseplate upper protein	1.50
phiMBL4	site-specific integrase	1.41
phiMBL4	hypothetical protein	1.37
phiMBL4	hypothetical protein	1.51
phiMBL4	hypothetical protein	2.01
phiMBL4	restriction endonuclease	1.71
phiMBL4	helix-turn-helix domain-containing protein	1.06
phiMBL4	DUF739 family protein	2.54
phiMBL4	phi PVL orf 32-like protein	1.82
phiMBL4	hypothetical protein	0.93
phiMBL4	hypothetical protein	2.76
phiMBL4	hypothetical protein	2.57
phiMBL4	hypothetical protein	3.65
phiMBL4	phage DNA-binding protein	2.55
phiMBL4	DUF1270 family protein	2.31
phiMBL4	hypothetical protein	2.05
phiMBL4	DUF1108 family protein	3.62
phiMBL4	DUF2483 domain-containing protein	3.65
phiMBL4	ATP-binding protein	3.18
phiMBL4	single-strand DNA-binding protein	3.51
phiMBL4	phi PV83 orf 19-like protein	3.74
phiMBL4	uncharacterized protein	3.56
phiMBL4	hypothetical protein	1.44
phiMBL4	phage conserved hypothetical protein	3.61

phiMBL4	AAA family ATPase	4.26
phiMBL4	hypothetical protein	3.29
phiMBL4	putative phi ETA-like protein	2.98
phiMBL4	DUF1064 domain-containing protein	3.53
phiMBL4	DUF3113 family protein	2.92
phiMBL4	PVL ORF-50-like family phage protein	3.66
phiMBL4	phage conserved Open Reading Frame 51	4.18
phiMBL4	phage protein DUF1024	4.19
phiMBL4	dUTP pyrophosphatase	4.77
phiMBL4	hypothetical protein	4.53
phiMBL4	DUF1381 domain-containing protein	4.07
phiMBL4	hypothetical PVL phage protein	3.04
phiMBL4	transcriptional activator RinB family protein	2.87
phiMBL4	unnamed protein product [Staphylococcus phage PVL]	1.73
phiMBL4	hypothetical protein	1.13
phiMBL4	DUF1514 domain-containing protein	1.30
phiMBL4	hypothetical protein	1.18
phiMBL4	hypothetical protein/phi PVL ORF 62 homologue	2.34
phiMBL4	phage endonuclease	2.75
phiMBL4	phage terminase small subunit P27 family	2.44
phiMBL4	phage terminase large subunit	1.75
phiMBL4	hypothetical protein/phi PVL ORF 3 homologue	1.07
phiMBL4	phage portal protein	1.85
phiMBL4	HK97 family phage prohead protease	1.59
phiMBL4	phage major capsid protein	2.83
phiMBL4	hypothetical protein	1.16
phiMBL4	phage head-tail adapter protein	2.10
phiMBL4	phage head-tail adapter protein	1.68
phiMBL4	hypothetical protein/phi PVL orf 12-like protein	1.67
phiMBL4	phage tail tape measure protein	1.17
phiMBL4	hypothetical protein	4.30
phiMBL4	hypothetical protein	1.53
phiMBL4	Panton-Valentine bi-component leukocidin subunit S	2.37
phiMBL4	Panton-Valentine bi-component leukocidin subunit F	2.14
SaPImbl6	terminase small subunit	2.04
SaPImbl6	pathogenicity island protein	1.74
SaPImbl6	spore coat protein	1.79
SaPImbl6	pathogenicity island 1 protein gp6	1.71
SaPImbl6	pathogenicity island protein	1.17
SaPImbl6	pathogenicity island protein	0.99
SaPImbl6	pathogenicity island protein	1.28
SaPImbl6	hypothetical protein	1.17

Table S4.
List of identified mutations of pyo^{R1-3} mutants

Mutant	Description	Mutation type	Position
pyo^{R1}	transcriptional regulator MarR family	Nonsense C to T	(60;[*:60])
	HD-domain containing protein	Missense G to A	(209;209)
	NAD(P)/FAD-dependent oxidoreductase	Deletion ACCTACACCG to A	(361;361)
	HAD family hydrolase	Missense TTA to T	(119;119)
	phage major capsid protein	Nonsense G to T	(179;[*:179])
	aquaporin family protein	Missense T to G	(145;145)
pyo^{R2}	FUSC family protein	Frameshift G to GT	(376;[*:394])
	transposase	Missense A to G	(50;50)
		Insertion T to C	(59;59)
	arsenic transporter	Missense T to C	(74;74)
		Missense A to C	(64;64)
		Missense G to T	(49;49)
	anthranilate phosphoribosyltransferase	Frameshift CCA to C	(77;[*:81])
	Methylenetetrahydrofolate-tRNA-(uracil(54)-C(5))-methyltransferase (FADH(2)-oxidizing) TrmFO	Missense A to T	(157;157)
	hypothetical protein	Frameshift TC to T	(43;[*:52])
		Missense C to T	(43;43)
	NAD(P)/FAD-dependent oxidoreductase	Nonsense C to A	(129;[*:129])
	peptidase T	Missense T to A	(94;94)
	YSIRK-type signal peptide-containing protein	Missense C to G	(1325;1325)
	YSIRK-type signal peptide-containing protein	Missense G to T	(851;851)<br>(847;847)<br>(835;835)<br>(841;841)
	tandem-type lipoprotein	Frameshift A to AT	(155;[*:156])
		Frameshift AT to A	(155;[*:178])
pyo^{R3}	Cof-type HAD-IIB family hydrolase	Missense G to A	(72;72)
	transcriptional regulator MarR family	Frameshift G to GA	(84;[*:85])
	transposase	Insertion T to C	(59;59)
	arsenic transporter	Missense T to C	(74;74)
		Missense A to C	(64;64)
		Missense G to T	(49;49)
		Missense A to G	(45;45)
	staphylococcal enterotoxin type M	Missense C to A	(117;117)
	aquaporin family protein	Missense T to A	(193;193)

hypothetical protein	Frameshift TC to T	(43;[*:52])
	Missense C to T	(43;43)
heme uptake protein IsdB	Deletion CCAA to C	(66;62)
NAD(P)/FAD-dependent oxidoreductase	Nonsense C to A	(129;[*:129])
ABC transporter permease	Frameshift AAT to A	(104;[*:106])
response regulator transcription factor	Frameshift A to AT	(168;[*:173])
	Frameshift AT to A	(168;[*:192])
PTS-dependent dihydroxyacetone kinase phosphotransferase subunit DhaM	Frameshift TC to T	(35;[*:52])
HAD family hydrolase	Missense TTA to T	(119;119)
YSIRK-type signal peptide-containing protein	Missense C to G	(1325;1325)
YSIRK-type signal peptide-containing protein	Missense A to T	(804;804)
	Missense G to T	(847;847)
		(841;841)
GntR family transcriptional regulator	Missense C to T	(44;44)
	Nonsense G to A	(227;[*:227])

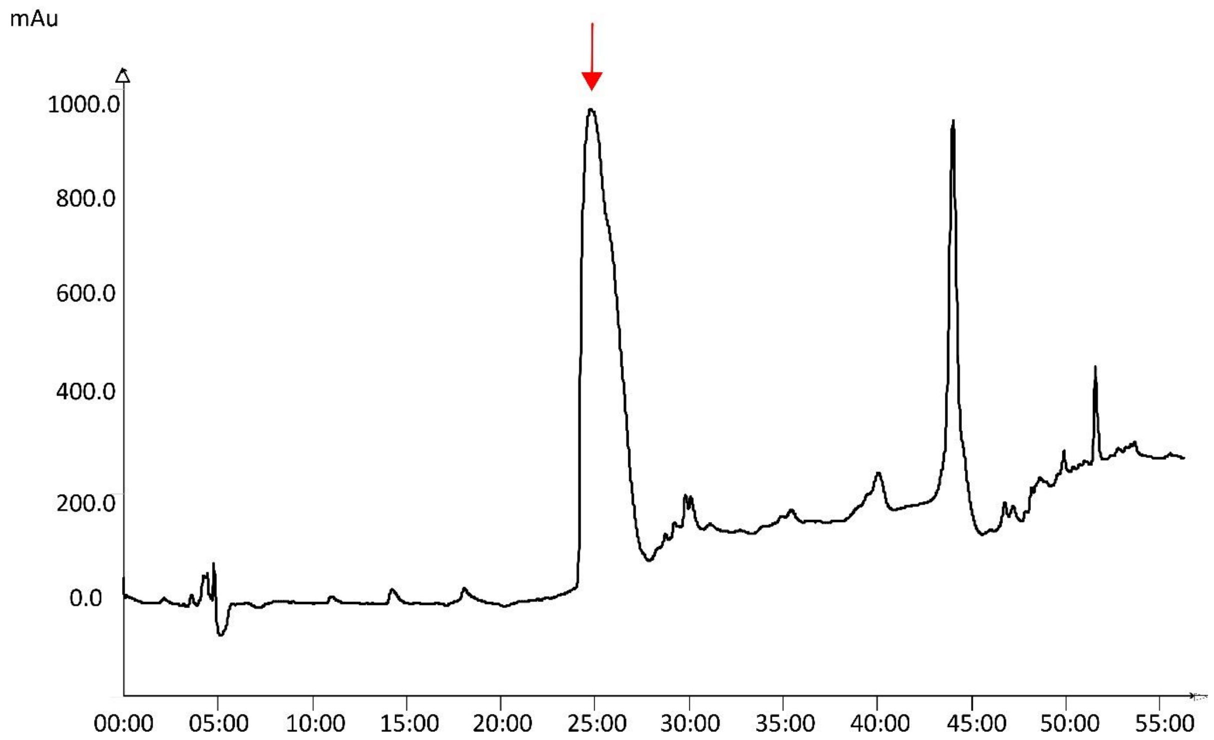

Fig. S1.

Preparative HPLC chromatogram from the pyocyanin purification. The elution peak of pyocyanin is indicated with red arrow.

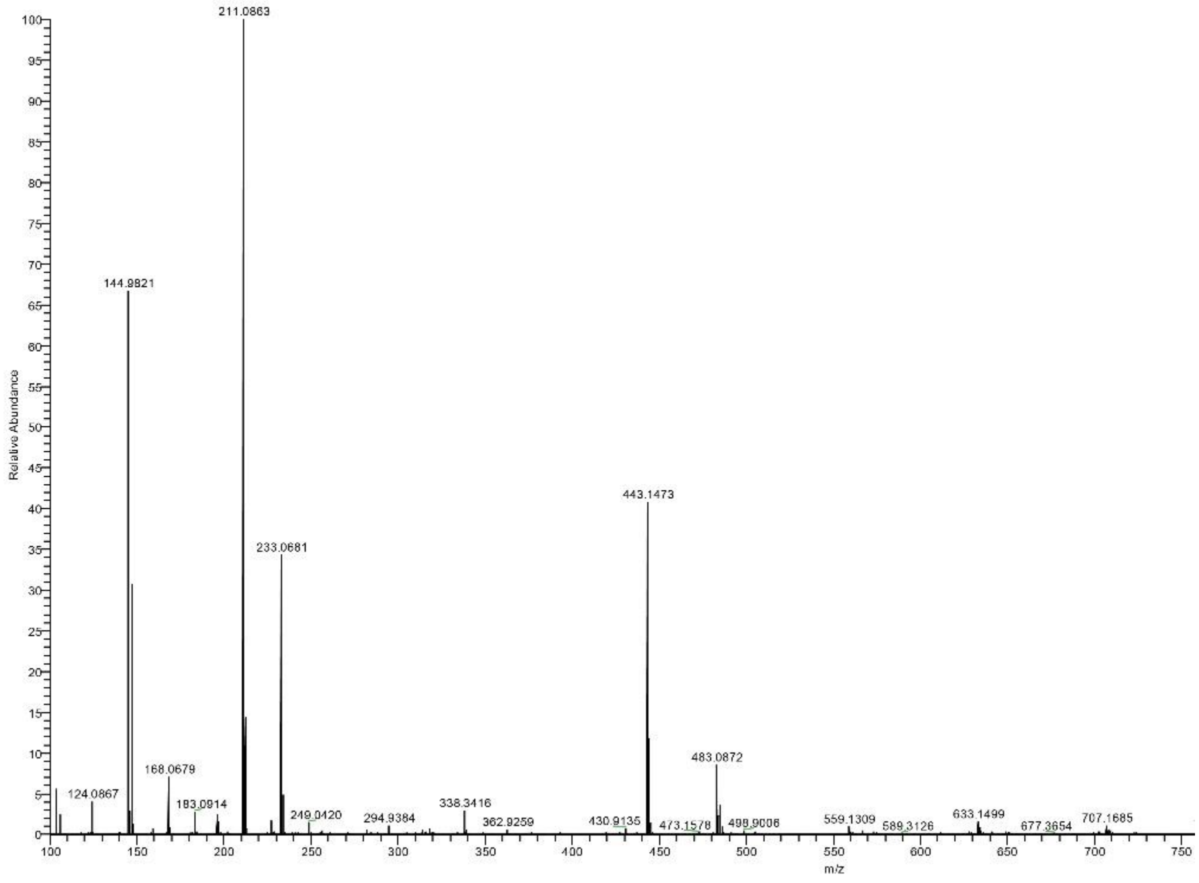

Fig. S2.
HRMS mass spectrum of isolated pyocyanin.

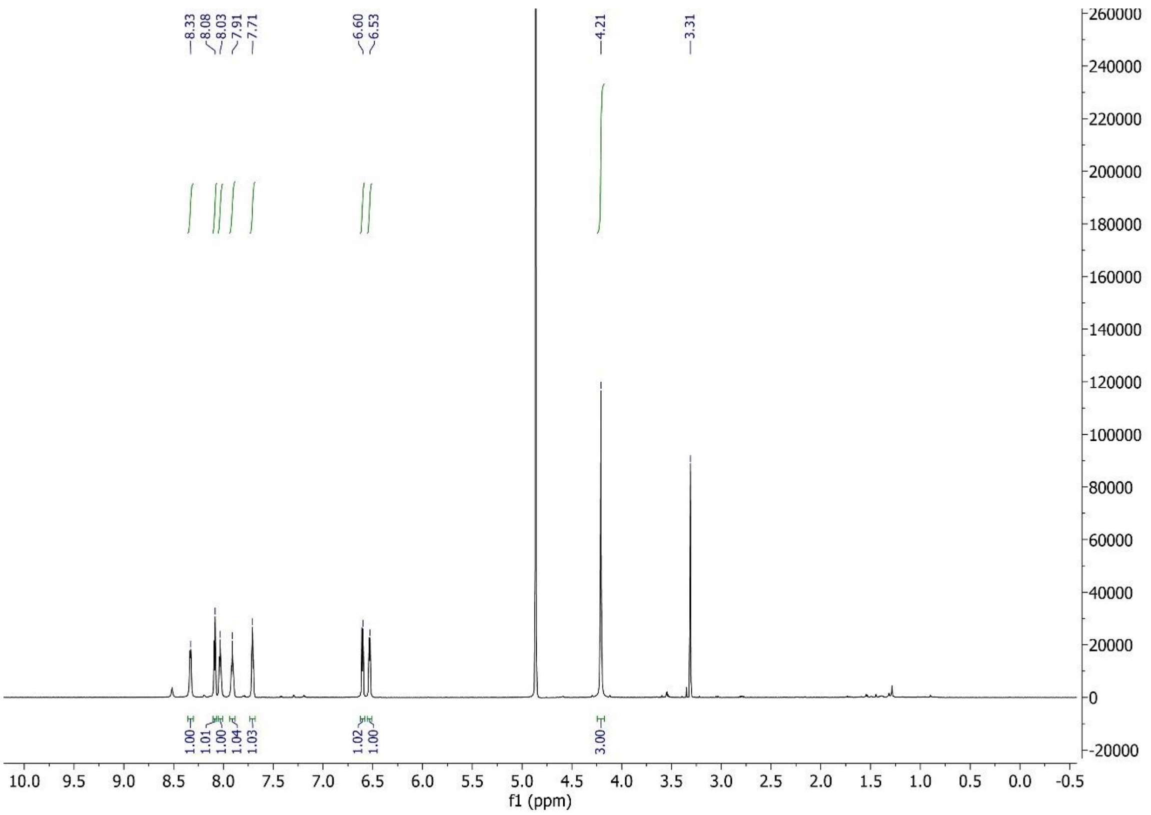

Fig. S3A.

^{1}H-NMR spectrum of isolated pyocyanin.

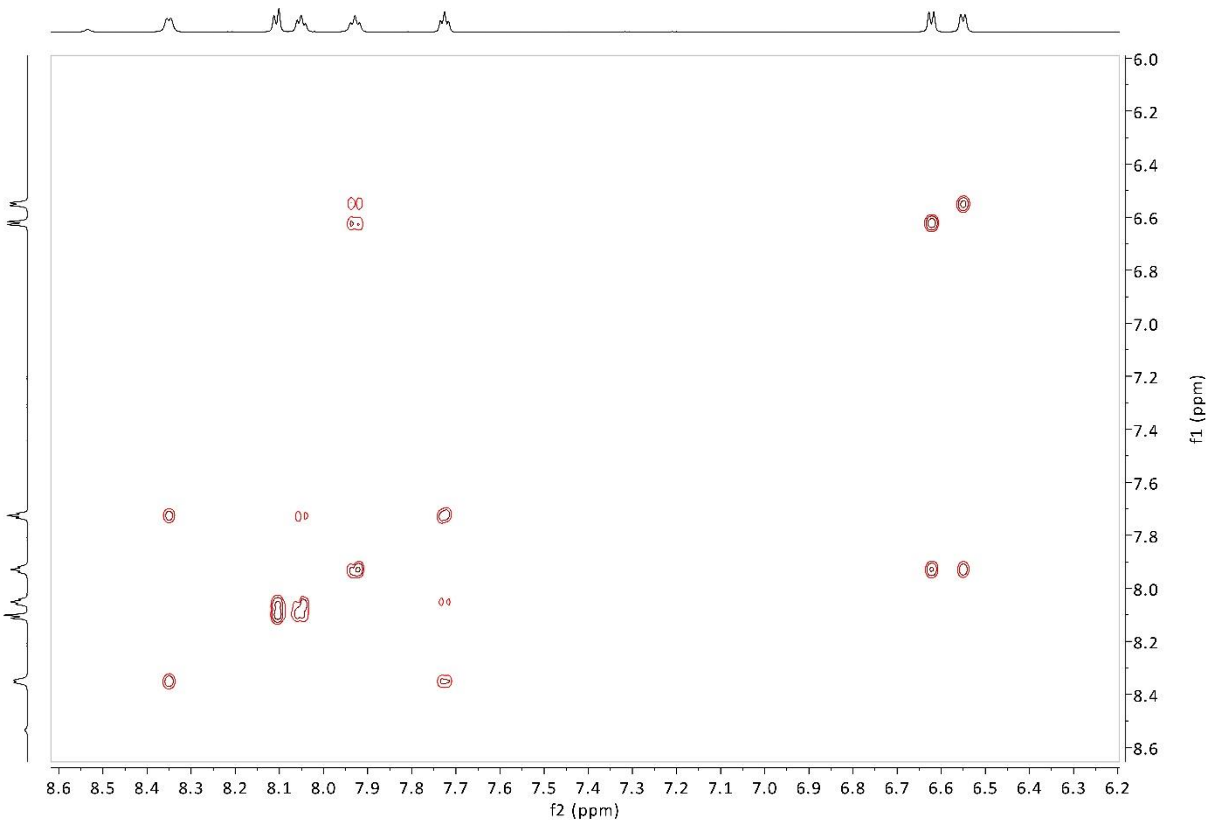

Fig. S3B.

COSY NMR spectrum of isolated pyocyanin.

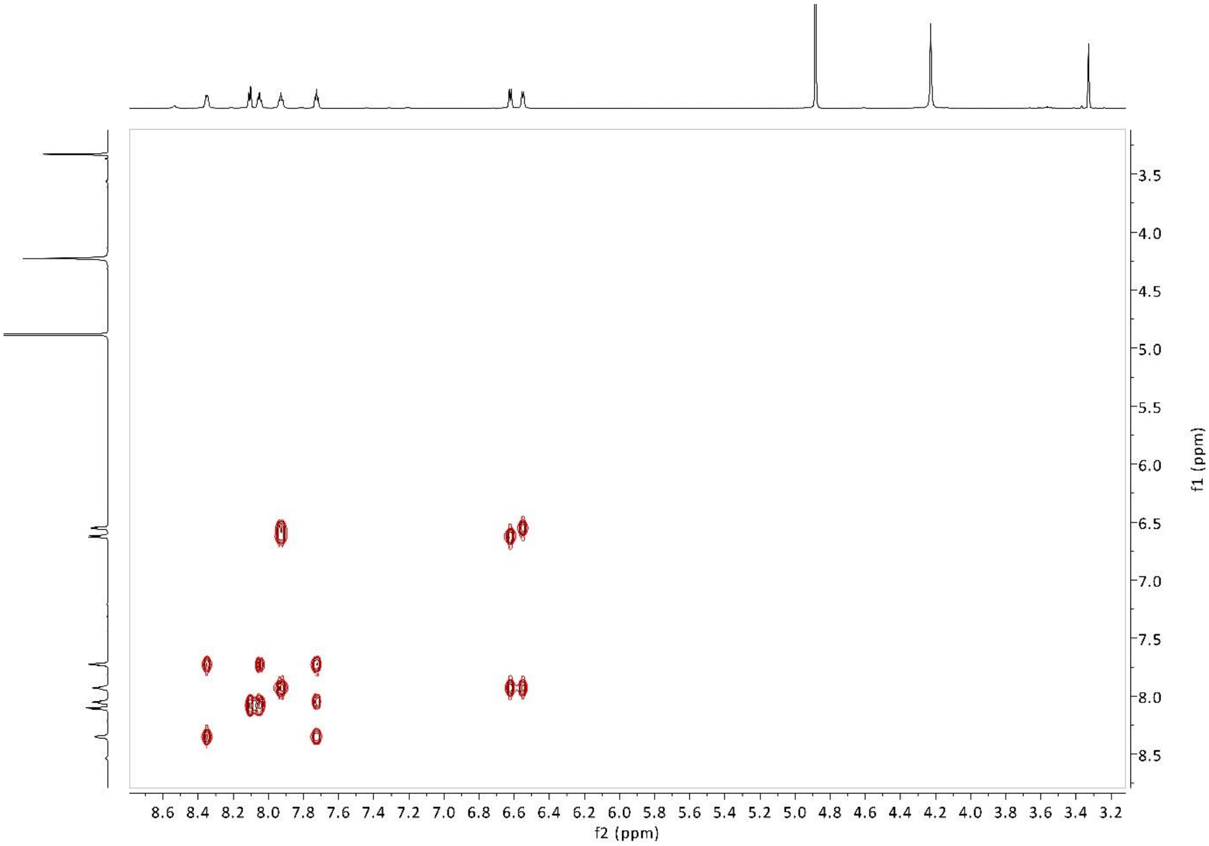

Fig. S3C.

HSQC NMR spectrum of isolated pyocyanin.

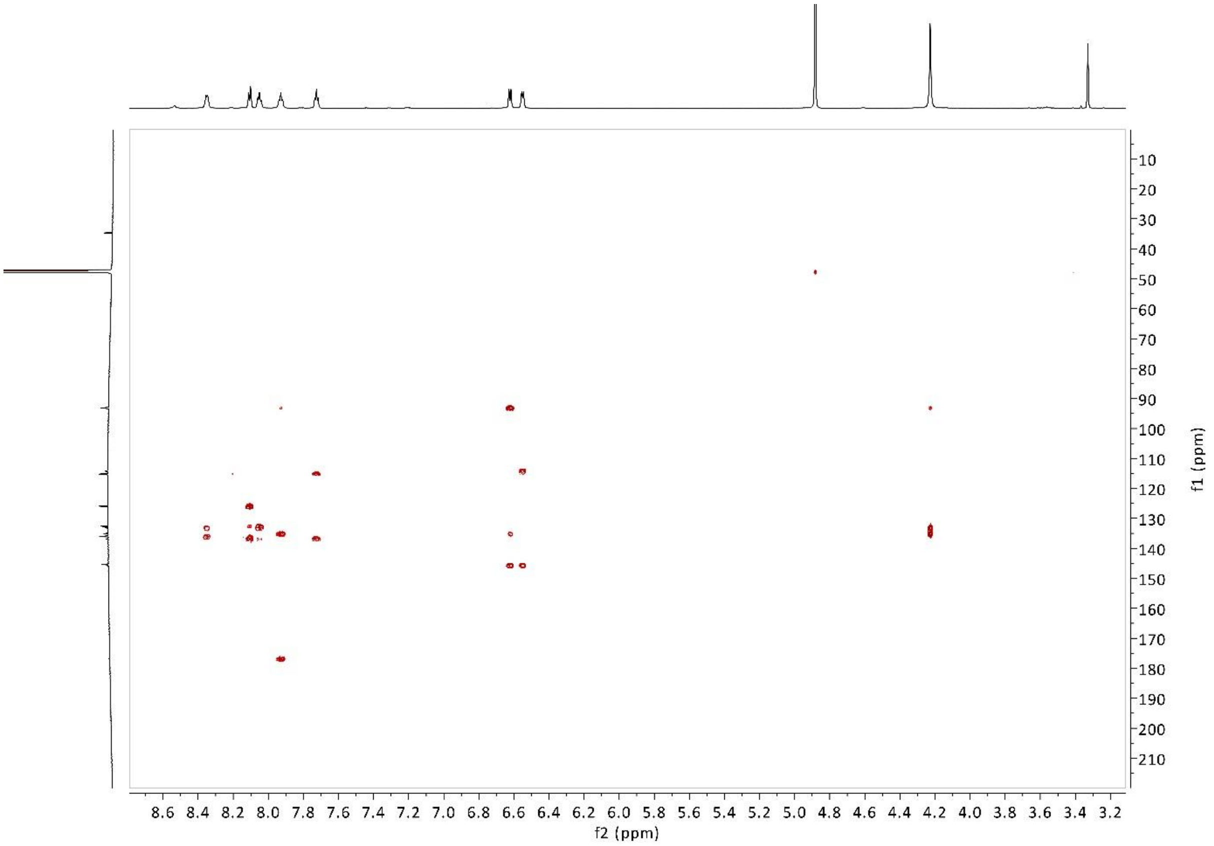

Fig. S3D.

HMBC NMR spectrum of isolated pyocyanin.

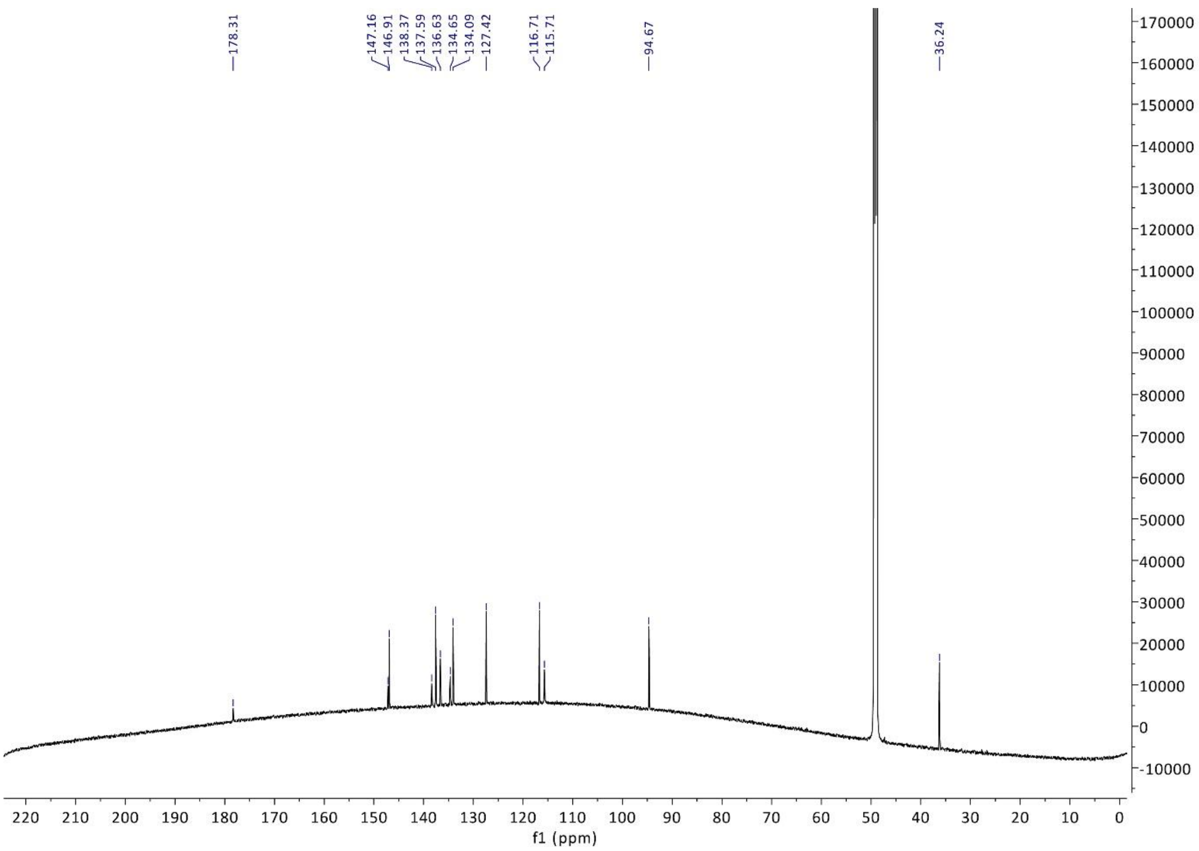

Fig. S3E.
^{13}C-NMR spectrum of isolated pyocyanin.

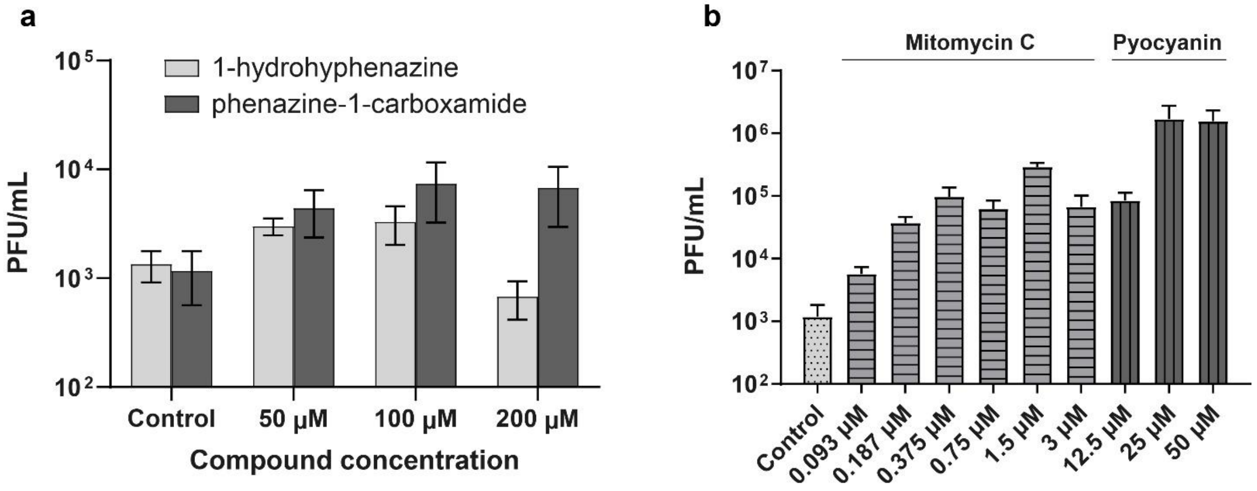

Fig. S4.

Pyocyanin is more a potent prophage inducer than 1-hydroxyphenazine and phenazine-1-carboxamide, and mitomycin C. a, Phage titre counts for phenazine-treated supernatants for *S. aureus* ATCC 6341 in CCY medium**.** Prior to induction, MIC values for each of the phenazines were determined. In addition to the MIC value of 200 µM, two lower concentrations (50 and 100 µM) were selected in the prophage induction assay. **b,** Prophage induction with mitomycin C and pyocyanin for *S. aureus* ATCC 6341 in CCY medium. The selection of the pyocyanin concentrations was based on the MIC value for pyocyanin (50 µM) and on the highest inducing concentration for mitomycin C (1.5 µM). **a** and **b**, In both experiments cells were induced at OD_{600} 0.8, the supernatants were harvested after 4 h incubation with the compounds and plaque assays were performed with *S. aureus* RN4220. Results are expressed as PFU/mL. For each compound three biologically independent replicates of phage induction assays were performed.

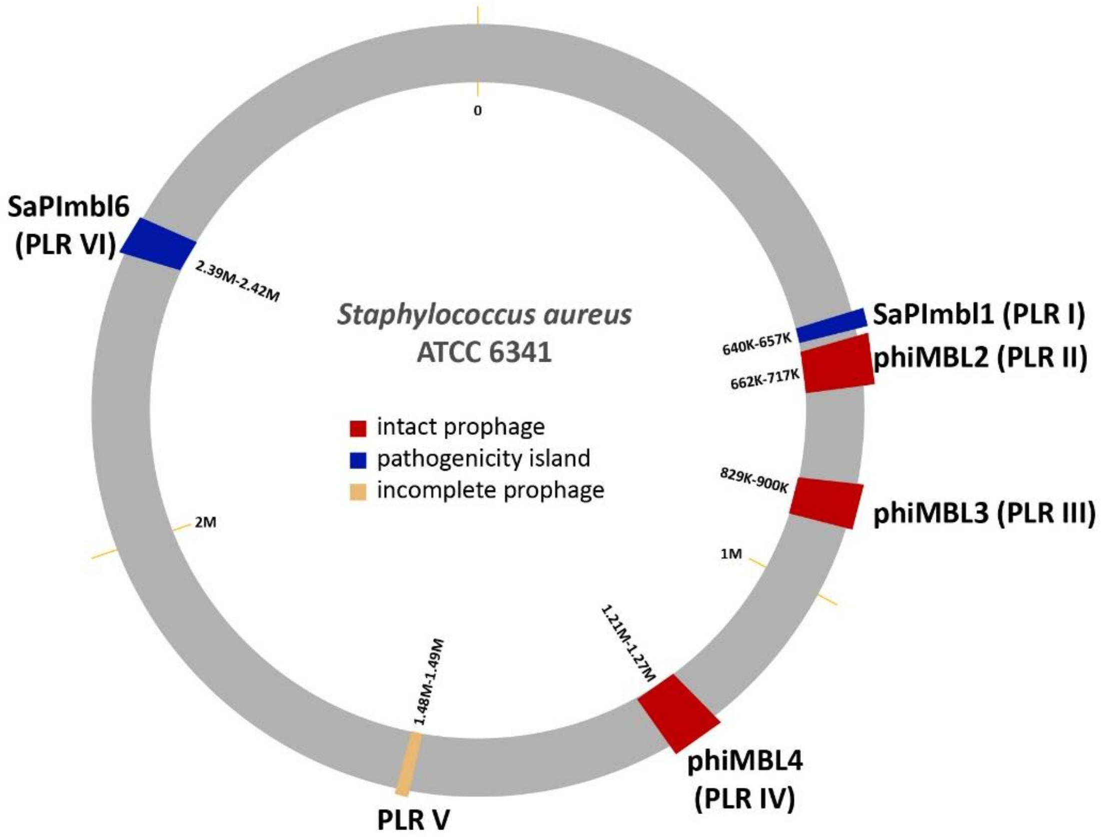

Fig. S5.

Genome of *S. aureus* ATCC 6341 with its prophage-like regions (PLRs) mapped by Phaster. Genomes for each of the PLRs were analyzed in the NCBI database (http://www.ncbi.nlm.nih.gov) with BlastN, BlastP and ORFfinder tools. PLRs II, III and IV (phiMBL2, phiMBL3 and phiMBL4) were characterized as intact prophages, PLRs I and VI (SaPImbl1 and SaPImbl6) as pathogenicity islands. PLR V was tentatively characterized as incomplete prophage.

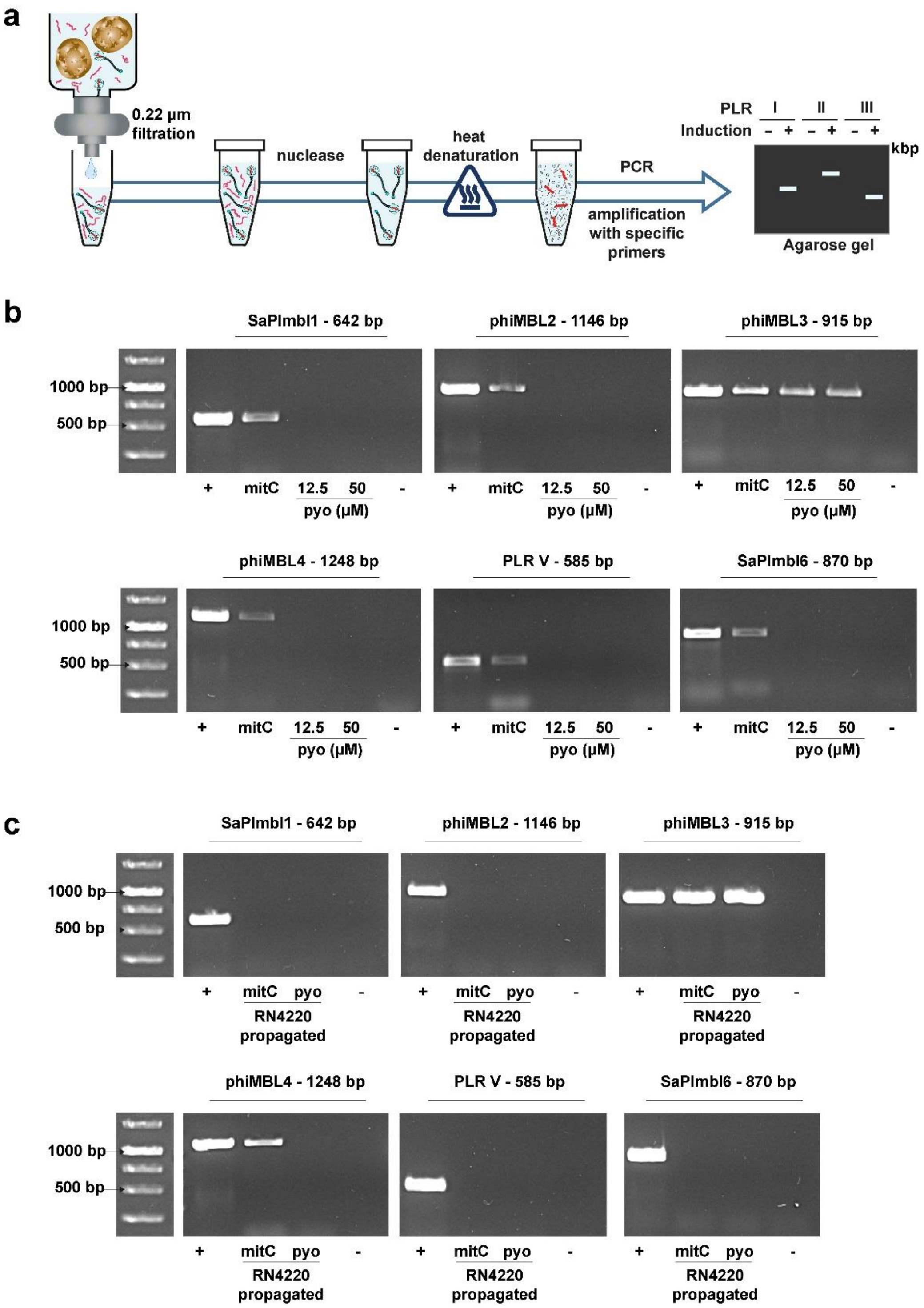

Fig. S6.

PCR-based detection of phage production. a, Schematic representation of the PCR-based detection method. Cells of *S. aureus* ATCC 6341 were grown to OD_{600} 0.8 and then incubated with the compounds for 4 h. The collected, sterile-filtered supernatants were DNase treated to digest the genomic DNA, then heated at 75˚C to inactivate the DNase and finally added to the PCR

mixture. The intact DNA packed in the phage capsids is released during a heat denaturation step prior to amplification. A prophage region was considered induced when the presence of a DNA band was detected by gel electrophoresis. In the experiments, the major capsid proteins of the prophages, distinctive pathogenicity island proteins and a phage scaffold protein (PLR V) were selected as diagnostic fingerprints. **b**, Agarose gels of PCR amplified samples from culture supernatants of *S. aureus* ATCC 6341 after treatment with 1.5 μM mitomycin C and pyocyanin (12.5 μM and 50 μM). DMSO-treated samples served as negative control in the assay and the extracted genome of *S. aureus* ATCC 6341 as positive control. **c**, Agarose gels for PCR-based detection of prophage-like elements successfully propagated in *S. aureus* RN4220. *S. aureus* ATCC 6341 was treated with 1.5 μM mitomycin C and 25 μM pyocyanin. Subsequently, prophage-like elements in culture supernatants were propagated on a lawn of *S. aureus* RN4220. After plaques developed on the bacterial lawn, plates were incubated with SM buffer overnight at 4˚C. The phage solution was precipitated with PEG/NaCl and the concentrated phages were collected with TMN buffer. DMSO-treated samples propagated in RN4220 served as negative control in the assay and the extracted genome of *S. aureus* ATCC 6341 as positive control. **b** and **c**, For each treatment five biological replicates were performed and representative results are shown.

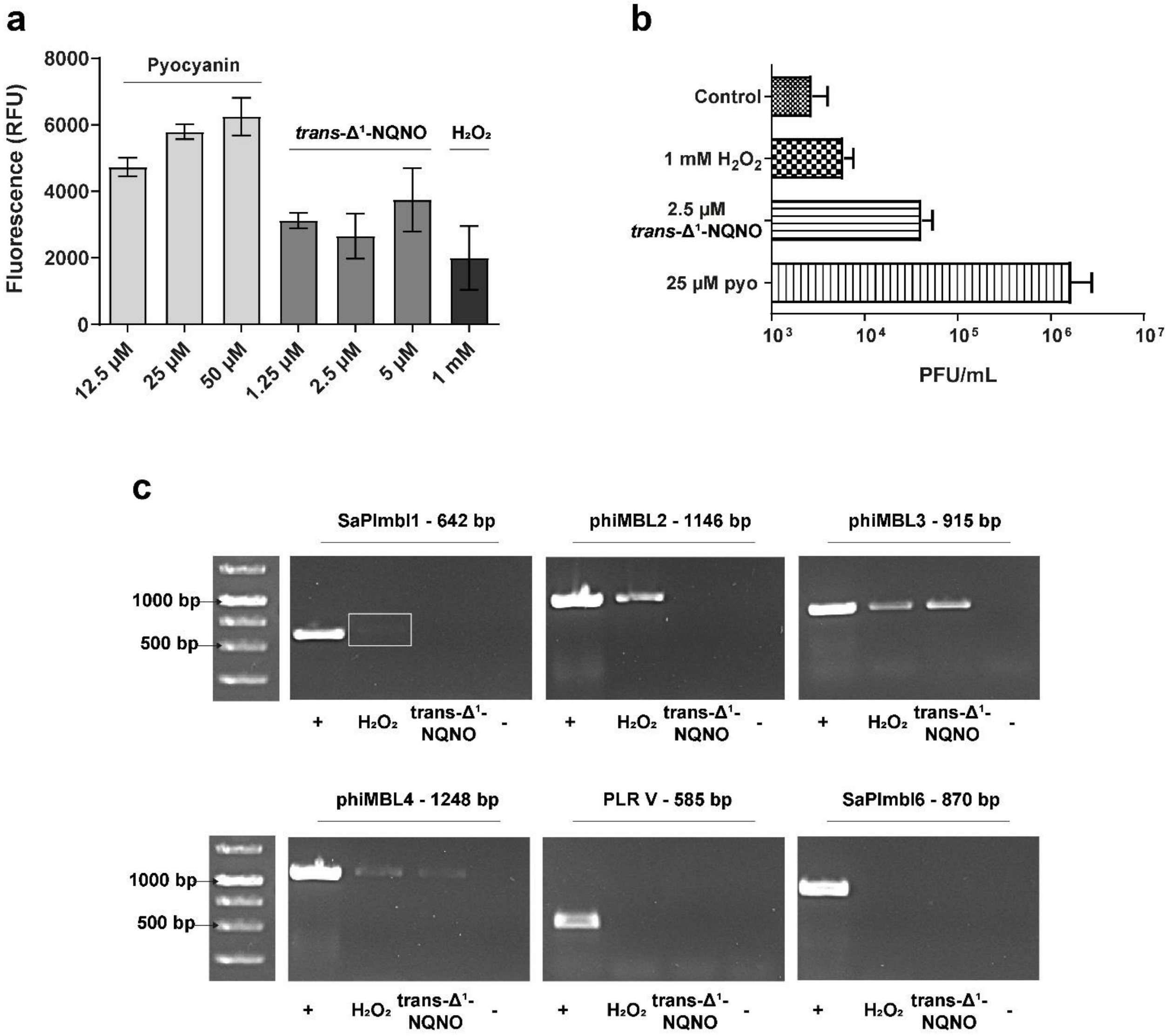

Fig. S7.

Pyocyanin-mediated ROS generation, prophage induction and selectivity differ from other respiratory toxins. a, Reactive oxygen species (ROS) detection with 2',7'-dichlorofluorescin diacetate (DCFDA). The initial cell count prior incubation with each of the compounds was adjusted to 4 x 10^8. Cells were treated with 20 µM DCFDA and incubated 2 h with the respective compounds. The measurements were performed in biological triplicates and the results presented were normalized to the fluorescence intensity of the control. **b**, Plaque forming unit (PFU/mL) counts for *S. aureus* ATCC 6341 treated with the highest-inducing concentrations of the three respiratory chain inhibitors in CCY medium. For each compound, three biological phage induction assay replicates were performed at the indicated concentrations. **c**, Agarose gels for samples treated with 1 mM H_2O_2 and 2.5 µM *trans*-Δ^1-NQNO. DMSO-treated samples served as negative control in the assay and the genome of ATCC 6341 as positive control. For each treatment five biological replicates were performed and representative results are shown.

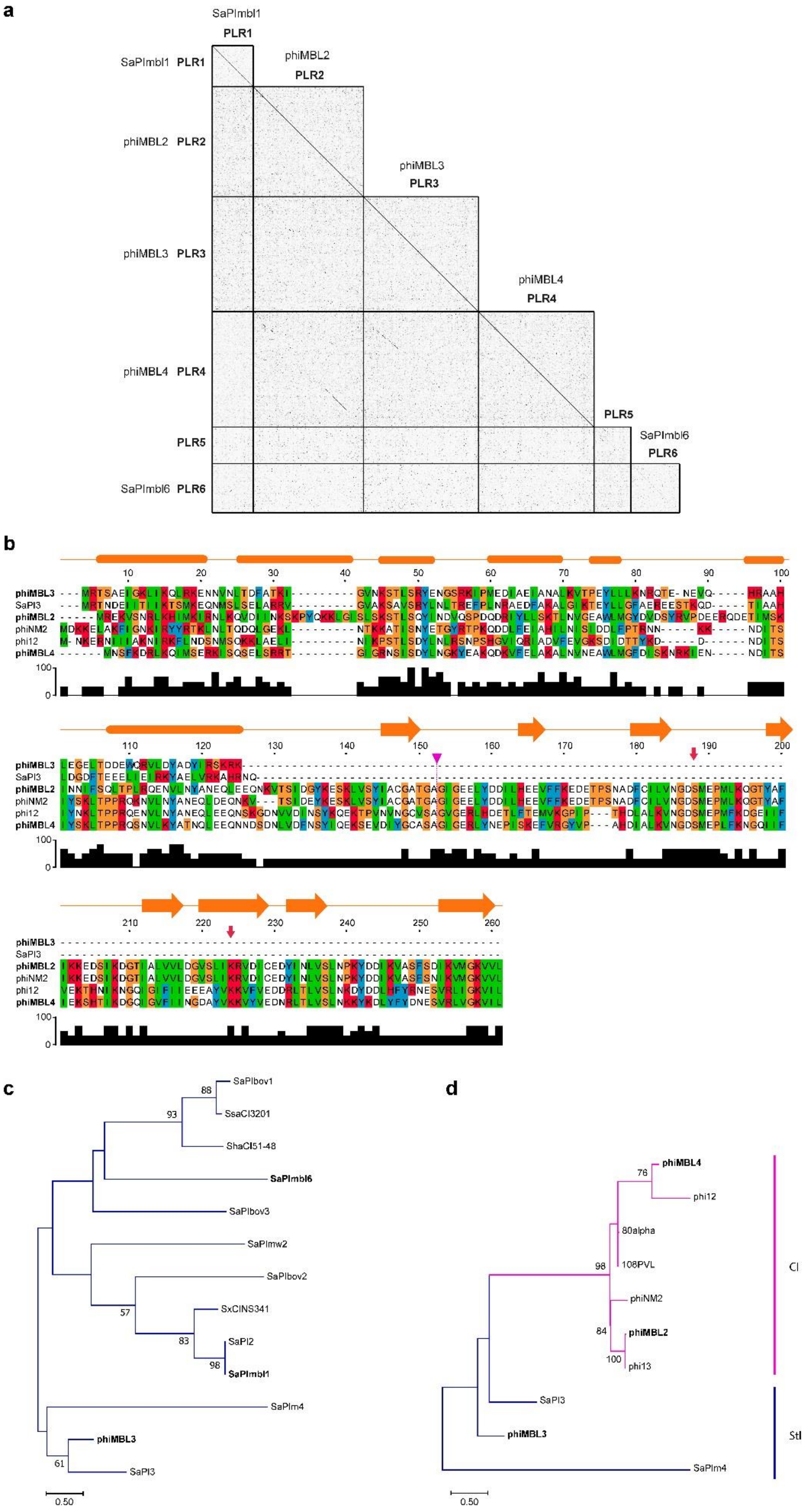
a
SaPImbl1 PLR1
phiMBL2 PLR2
phiMBL3 PLR3
phiMBL4 PLR4
PLR5
SaPImbl6 PLR6
b
phiMBL3
SaPI3
phiMBL2
phiNM2
phi12
phiMBL4
c
SaPIbov1
SsaCI3201
ShaCI51-48
SaPImbl6
SaPIbov3
SaPImw2
SaPIbov2
SxCINS341
SaPI2
SaPImbl1
SaPIm4
phiMBL3
SaPI3
0.50
d
phiMBL4
phi12
80alpha
108PVL
phiNM2
phiMBL2
phi13
SaPI3
phiMBL3
SaPIm4
CI
StI
0.50

Fig. S8.

Prophage phiMBL3 harbours a truncated repressor phylogentically related to Stl repressors of SaPIs. **a**, Dotplots displaying genomic sequence identities between different prophage-like regions (PLRs) were generated with the program Genome Pair Rapid Dotter, Gepard 1.40 (http://cube.univie.ac.at/gepard) using a word length of 9 and a window size of 0. **b**, Sequence alignments of CI-like and Stl-like repressor homologs of the three prophages phiMBL2, phiMBL3, and phiMBL4 with the closely related prophages (phi12 and phiNM2) and a SaPI (SaPI3) performed with PRALINE with the color schemes adapted from CLUSTALX. Secondary structure predictions (orange) with DSSP and PSIPRED are indicated as β-sheets (tubes) and α-helices (arrows). Active site residues are labelled by arrows (red) and the autocleavage site by a triangle (pink). **c**, Phylogenetic trees of Stl-like repressor homologs of SaPIs and **d**, CI-like repressor homologs of prophages (pink) and Stl-like repressor homologs of SaPIs (blue). Trees were constructed as maximum likelihood trees with bootstrap test (500 replications) using MEGA-X. Bootstrap values >50 are indicated at the corresponding nodes.

References

1. D. W. Essar, L. Eberly, A. Hadero, I. P. Crawford, Identification and characterization of genes for a second anthranilate synthase in Pseudomonas aeruginosa: interchangeability of the two anthranilate synthases and evolutionary implications. *Journal of bacteriology* **172**, 884-900 (1990).
2. M. Mirande, M. Lazard, J. P. Waller, Small-scale purification of bacteriophage lambda DNA by an airfuge centrifugation step in cesium chloride gradients. *Gene Anal Tech* **5**, 80-82 (1988).
3. J. Krumsiek, R. Arnold, T. Rattei, Gepard: a rapid and sensitive tool for creating dotplots on genome scale. *Bioinformatics* **23**, 1026-1028 (2007).
4. V. A. Simossis, J. Heringa, PRALINE: a multiple sequence alignment toolbox that integrates homology-extended and secondary structure information. *Nucleic acids research* **33**, W289-294 (2005).
5. W. Kabsch, C. Sander, Dictionary of protein secondary structure: pattern recognition of hydrogen-bonded and geometrical features. *Biopolymers* **22**, 2577-2637 (1983).
6. D. T. Jones, Protein secondary structure prediction based on position-specific scoring matrices. *J Mol Biol* **292**, 195-202 (1999).
7. D. Arndt *et al.*, PHASTER: a better, faster version of the PHAST phage search tool. *Nucleic acids research* **44**, W16-21 (2016).
8. Y. Zhou, Y. Liang, K. H. Lynch, J. J. Dennis, D. S. Wishart, PHAST: a fast phage search tool. *Nucleic acids research* **39**, W347-352 (2011).
9. M. J. Noto, W. J. Burns, W. N. Beavers, E. P. Skaar, Mechanisms of Pyocyanin Toxicity and Genetic Determinants of Resistance in *Staphylococcus aureus*. *Journal of bacteriology* **199**, (2017).
